# BRD2 promotes antibody class switch recombination by facilitating DNA repair in collaboration with NIPBL

**DOI:** 10.1093/nar/gkae204

**Published:** 2024-04-03

**Authors:** Santosh K Gothwal, Ahmed M Refaat, Mikiyo Nakata, Andre Stanlie, Tasuku Honjo, Nasim A Begum

**Affiliations:** Department of Immunology and Genomic Medicine, Kyoto University Graduate School of Medicine, Kyoto 606-8501, Japan; Department of Immunology and Genomic Medicine, Kyoto University Graduate School of Medicine, Kyoto 606-8501, Japan; Center for Cancer Immunotherapy and Immunobiology, Kyoto University Graduate School of Medicine, Kyoto 606-8501, Japan; Zoology Department, Faculty of Science, Minia University, El-Minia 61519, Egypt; Department of Immunology and Genomic Medicine, Kyoto University Graduate School of Medicine, Kyoto 606-8501, Japan; Center for Cancer Immunotherapy and Immunobiology, Kyoto University Graduate School of Medicine, Kyoto 606-8501, Japan; Department of Immunology and Genomic Medicine, Kyoto University Graduate School of Medicine, Kyoto 606-8501, Japan; Department of Immunology and Genomic Medicine, Kyoto University Graduate School of Medicine, Kyoto 606-8501, Japan; Center for Cancer Immunotherapy and Immunobiology, Kyoto University Graduate School of Medicine, Kyoto 606-8501, Japan; Department of Immunology and Genomic Medicine, Kyoto University Graduate School of Medicine, Kyoto 606-8501, Japan; Center for Cancer Immunotherapy and Immunobiology, Kyoto University Graduate School of Medicine, Kyoto 606-8501, Japan

## Abstract

Efficient repair of DNA double-strand breaks in the Ig heavy chain gene locus is crucial for B-cell antibody class switch recombination (CSR). The regulatory dynamics of the repair pathway direct CSR preferentially through nonhomologous end joining (NHEJ) over alternative end joining (AEJ). Here, we demonstrate that the histone acetyl reader BRD2 suppresses AEJ and aberrant recombination as well as random genomic sequence capture at the CSR junctions. BRD2 deficiency impairs switch (S) region synapse, optimal DNA damage response (DDR), and increases DNA break end resection. Unlike BRD4, a similar bromodomain protein involved in NHEJ and CSR, BRD2 loss does not elevate RPA phosphorylation and R-loop formation in the S region. As BRD2 stabilizes the cohesion loader protein NIPBL in the S regions, the loss of BRD2 or NIPBL shows comparable deregulation of S-S synapsis, DDR, and DNA repair pathway choice during CSR. This finding extends beyond CSR, as NIPBL and BRD4 have been linked to Cornelia de Lange syndrome, a developmental disorder exhibiting defective NHEJ and Ig isotype switching. The interplay between these proteins sheds light on the intricate mechanisms governing DNA repair and immune system functionality.

## Graphical Abstract

**Graphical Abstract:**
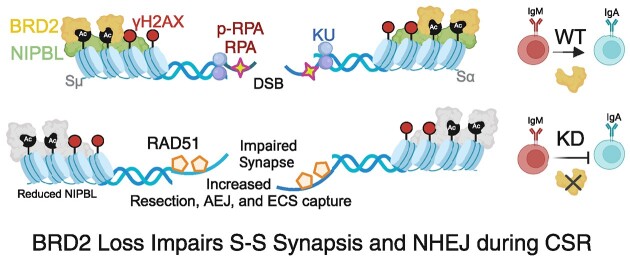

## Introduction

Antibody class switch recombination (CSR) supports the adaptive immune system by diversifying antibody effector functions, providing a way to fine-tune the immune responses to invading pathogens by increasing specificity and effectiveness. CSR involves precise recombination between two switch (S) regions of the immunoglobulin heavy chain locus (*IgH*), which enables B cells to switch from producing one isotype of antibody to another in response to different antigens (1). The CSR process is complex and requires precise regulation to ensure accurate recombination at the immunoglobulin heavy chain (*IgH*) locus and prevent genomic instability (2,3).

Activation-induced cytidine deaminase (AID) plays a critical role in the initiation of CSR by introducing double-stranded DNA breaks (DSBs) in the donor and acceptor switch (S) regions of the *IgH* locus (4,5). DNA breaks generated by AID must be repaired by classical nonhomologous end joining (C-NHEJ) to allow S−S recombination to minimize aberrant genomic rearrangement (6,7). However, studies have shown that CSR can occur in the absence of C-NHEJ DNA repair factors, such as Ku70/Ku80 and Ligase IV, through an alternative end joining (A-EJ) pathway that repairs DSBs through microhomology (MH) and is less efficient for CSR (8,9). The mechanism by which C-NHEJ promotion and suppression of A-EJ occurs to promote efficient CSR is not well understood. Emerging studies suggest that dysregulation of switch region chromatin remodeling, RNA/DNA hybrid or R-loop formation, and even switch germline transcript (GLT) processing can skew the repair pathway toward A-EJ and impair CSR (10–14). The underlying cause is not necessarily always a compromised DNA damage response (DDR) or impaired C-NHEJ factor assembly. The altered quality and proportion of DSBs due to structural perturbation of the target zones or limited AID loading can also divert the pathway toward A-EJ (13–15). More recently, RNA binding proteins, noncoding RNAs, cellular dNTP balance, and liquid−liquid phase separation involving repair condensate formation were also shown to play roles in S−S recombination and CSR (11,13,14,16,17). Collectively, these studies suggest that several regulatory cascades upstream of DDR work together to ensure proper CSR and prevent harmful genetic changes.

The chromatin environment and its regulation surrounding the DSB are crucial in generating an effective DDR to promote DNA repair (18,19). Histone acetylation helps attract chromatin readers and DSB repair proteins to the site of DNA double-strand breaks, promoting efficient DNA repair. Upon induction of CSR, histone acetylation is elevated in the recombining S regions (12,20,21), suggesting that the chromatin region is more accessible and favorable for DNA repair. The acetyl histone reader protein BRD4, a member of the bromodomain and extraterminal (BET) protein family, has been shown to promote C-NHEJ-mediated recombination through 53BP1 and other repair protein recruitment in the S region (12,22). BRD4 depletion also decreased AID-induced *IgH/c-Myc*chromosomal translocation, often associated with Burkitt lymphoma and facilitated by the C-NHEJ pathway (23,24). Accordingly, BRD4-mediated C-NHEJ repair was also found to be responsible for TMPRSS2/ERG chromosomal translocation in prostate cancer (25). Additionally, BRD4 has been reported to be involved in homologous recombination (HR) and in the regulation of insulated chromatin domain formation near the site of DSB (26–28). Intriguingly, BRD4 has been implicated in the neurodevelopmental disorder Cornelia de Lange Syndrome (CdLS) (29,30), typically caused by mutations in the cohesion complex gene NIPBL. Generally, the CdLS phenotype is considered to be the consequence of super-enhancer defects, leading to transcriptional dysregulation (31). However, analysis of the CdLS-associated BRD4 mutation (Y430C) revealed a previously unrecognized DNA damage response defect (30). The Y430C mutation in the acetyl histone binding domain of BRD4 caused an intensified and prolonged DNA damage response in patient-derived cells, as observed in some NIPBL-associated CdLS (30,32,33). The paused or delayed DNA repair response suggests that BRD4 is a crucial genomic integrity regulator capable of eliciting diverse responses to DNA damage in a context-dependent manner.

Our current understanding of how other BRD proteins of the BET family, such as BRD2, BRD3 and BRDT, affect DNA repair and/or AID-induced genome instability in B cells is limited. The present study shows that BRD2 is another critical BET member that contributes to CSR, independent of BRD4, to promote S−S recombination through C-NHEJ. In activated B cells, it has been noted that BRD2 exhibits a more robust interaction with NIPBL than BRD4. This association aligns with their stability in the S region and the functional similarities shared between NIPBL and BRD2 but not with BRD4. Thus, the BRD2/NIPBL axis regulates several critical steps in CSR, including DNA break-end synapses, RPA phosphorylation and suppression of A-EJ and ECS insertion at CSR junctions. Although the association of NIPBL with CSR, CdLS and NHEJ was previously reported, the underlying mechanism remains unclear (34,35). The present study highlights the complexity of parallel and interwoven NHEJ repair pathways involving BRD2, BRD4 and NIPBL. This has potential implications for understanding the misregulated repair mechanisms associated with CSR-linked diseases such as CdLS and genomic instability in cancer involving BET and cohesin proteins (34,36–38).

## Materials and methods

### Culture of CH12F3-2A cells and stimulation for CSR

The mouse B-cell lymphoma line CH12F3-2A expressing *Bcl2* (39) was cultured in complete RPMI supplemented with 5% NCTC, 10% FBS and 0.05% 2-mercaptoethanol as described. For IgM to IgA isotype switch analysis, CH12F3-2A cells were stimulated with CIT (CD40-L, IL-4 and TGF-β) and harvested after 24 or 48 hours of induction as previously described (40). Cells were surface stained with FITC-conjugated anti-IgM (eBioscience) and PE-conjugated anti-IgA (Southern Biotechnology) antibodies. Flow cytometric analysis was performed using FACS-Calibur and CellQuest software (BD Biosciences). The antibodies used for staining are shown in Supplementary Table S1.

### Primary B-cell culture and stimulation for CSR

B lymphocytes were isolated from 6- to 8-week-old wild-type C57BL/6 mice using a mouse B lymphocyte enrichment kit (BD Biosciences). Naïve B cells were cultured in RPMI medium supplemented with 10% FBS, 1× nonessential amino acids, 1 mM sodium pyruvate, and 0.05% 2-mercaptoethanol. To induce IgG1 switching, primary B cells were stimulated with LPS and IL4 for 5 days. When we performed siRNA electroporation, we preactivated the primary culture with LPS for 2 days prior to siRNA introduction, followed by LPS and IL4 stimulation 6 h after transfection and continued culture for 3 days. Subsequently, these cells were stained with biotinylated anti-IgG1 in conjunction with allophycocyanin-labeled streptavidin (41,42). Flow cytometric analysis was performed using FACS-Calibur and CellQuest software (BD Biosciences).

### Gene knockdown and CSR rescue assays

**Figure 1.**
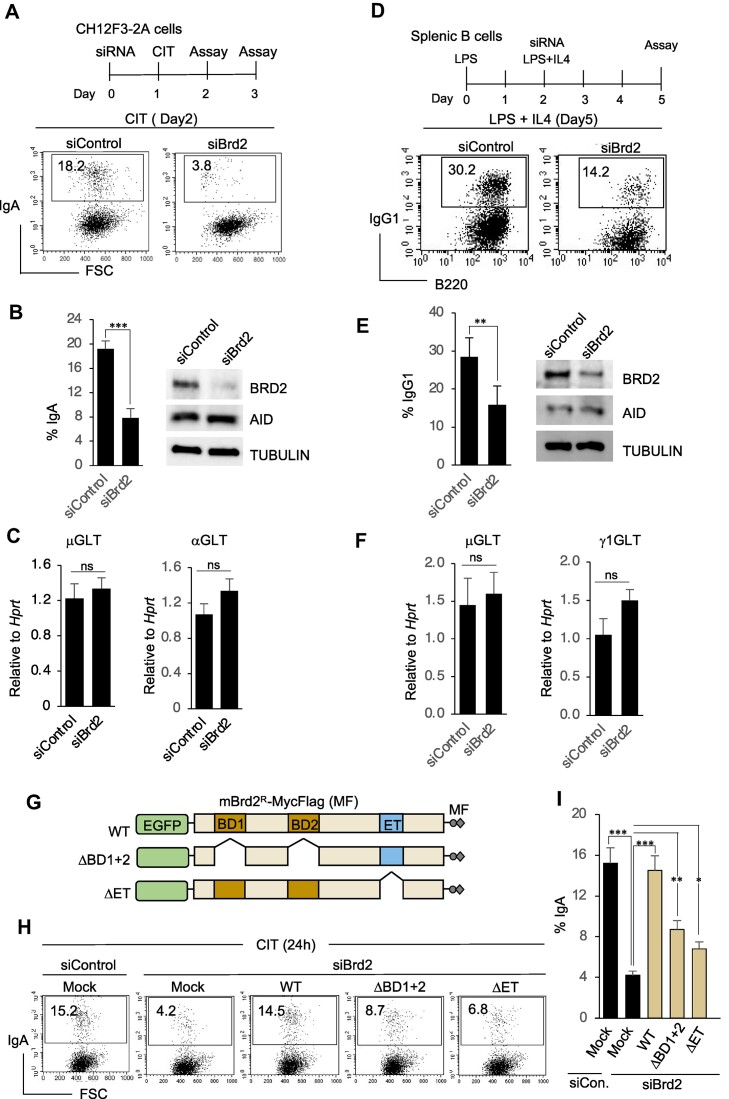
BRD2 is required for efficient immunoglobulin class switch recombination. (A–C) Effect of *Brd2* knockdown on CSR and GLT in CH12F3-2A cells. (**A**) FACS analysis of surface IgA expression in activated CH12F3-2A cells transfected with control siRNA (siControl) or siRNA against *Brd2* (siBrd2). The CSR to IgA was induced by CIT (CD40L, IL-4 and TGF-β) stimulation and harvested for the assays as depicted in the upper panel. (**B**) Bar plots show the compilation of IgA switching from three independent experiments. Western blot analysis of BRD2, AID, and loading control from whole cell extracts are shown next. (**C**) Quantitative RT−PCR analysis of μGLT and αGLT using total RNA isolated from the indicated samples. (D–F) Effect of *Brd2* knockdown on CSR and GLT in primary B cells. (**D**) FACS analysis of surface IgG1 expression in siControl- and siBrd2-transfected primary B cells stimulated with LPS and IL-4. CSR to IgG1 induction, and the assay timeline is shown in the top panel. (**E**) Bar plots show a compilation of IgG1 switching from two independent experiments. Western blot analysis of BRD2, AID, and loading control from whole cell extracts of activated primary B cells. (**F**) Quantitative RT−PCR analysis of μGLT and γ1GLT using total RNA isolated from the indicated siRNA-treated primary B cells. (**G–I**) CSR complementation by expressing wild-type (WT) or mutant BRD2 (ΔBD1 + 2 and ΔET) constructs in BRD2-depleted CH12F3-32A cells. (**G**) Schematics of Myc-Flag epitope-tagged *Brd2* constructs co-transfected with siBrd2 in (H). The ‘R’ superscript indicates that the transcripts produced from the constructs are resistant to siBrd2-mediated degradation. (**H**) Representative FACS profile of the CSR complementation assay. (**I**) The bar plots show CSR rescue efficiency by WT and BRD2 mutants calculated from three independent experiments. (B, C, E, F and I) The plot values represent the mean ± SD (*n* = 3 or 2 experiments). Statistical analysis was performed using Student's *t*-test (*P ≤ 0.05; **P ≤ 0.01; ***P ≤ 0.001; ns, non-significant).

To knock down a target gene in CH12F3-2A cells, we introduced 40 pmoles of chemically modified Stealth siRNA oligonucleotide (Thermo Fisher) into approximately 1 × 10^6^ cells by electroporation. The 96-well Lonza Nucleofector electroporation system and the SF Cell Line 96-well nucleofector kit (Lonza #V4SC-2096) were used to introduce siRNA or plasmid into CH12 F3-2A cells using the Nucleofector program # CM-150. One day later, the cells were stimulated by CIT and cultured for another 1–2 days before harvest and downstream analysis. For the CSR complementation assay, siRNA and 1–1.5 μg of EGFP fused mBrd2^R^-MF construct (Figure 1G) were co-electroporated into CH12F3-2A cells. To induce CIT-independent CSR in CH12F3-2A cells, Sμ- and Sα-specific CRISPR/Cas9 constructs were co-electroporated with siControl or siBrd2 as needed. To deliver siRNA into the primary B cells, ∼6–7 × 10^5^ LPS preactivated B cells mentioned were subjected to electroporation using a mouse B-cell Nucleofector Kit and the program # 96-DI-100. Supplementary Table S2 contains necessary information on the siRNAs used.

### Immunoblotting

A routine immunoblotting protocol was adopted throughout the experiments. Briefly, we lysed 2 × 10^6^ cells in RIPA buffer (300 mM Tris–HCl pH 7.4, 150 mM NaCl, 10% glycerol, 1% Triton X-100, 0.05% sodium deoxycholate and 5 mM EDTA) containing 1x protease inhibitor (EDT free). Cell lysates were clarified by centrifugation at 15 000 rpm for 10 min at 4°C. For gel electrophoresis, protein samples were prepared in 1x SDS sample buffer containing β-mercaptoethanol and heated at 85°C for 10 min. The samples were electrophoresed in 4–20% precast gel (Mini Protein TGX™, Bio-Rad), transferred to a nitrocellulose membrane and processed for Western blot analysis and ECL (Pierce) detection following standard protocols. Antibodies are listed in Supplementary Table S1.

### RT−qPCR

Total RNA was extracted from B cells using TRIzol (Gibco BRL) and subsequently purified with the RNeasy MinElute Cleanup Kit (QIAGEN). cDNA synthesis was achieved using Oligo dT primers and Invitrogen SuperScript IV Reverse Transcriptase. Quantitative PCR (qPCR) was conducted using PowerUp SYBR Green Master Mix (Thermo Fisher Scientific) and carried out in duplicate or triplicate on Applied Biosystems real-time PCR instrument. Data analysis was performed by calculating ΔCq values normalized to *Hprt* gene expression, adhering to the Minimum Information for Publication of Quantitative Real-Time PCR Experiments (MiQE) guidelines (43). RT-qPCR primer sequences and the MIQE checklist are provided in Supplementary Table S2 and Supplementary Table S4, respectively.

### ChIP assay

ChIP assays were conducted using the ChIP-IT® Express Kit (Active Motif) following the manufacturer's protocol. Upon CIT stimulation, siRNA-transfected CH12F3-2A cells (∼5 × 10^6^) were fixed in 1% formaldehyde for 8 min at room temperature, with crosslinking termination achieved using 0.125 M glycine. Subsequently, crosslinked cells were lysed, and nuclei were resuspended in 350 μl of shearing buffer, with DNA fragments targeted for fragmentation between 200–500 bp using a Bioruptor (Diagenode).

Approximately 80 μl of chromatin was incubated for each IP with 2–3 μg of the selected antibody, followed by overnight rotation at 4°C with protein A/G magnetic beads. The bead-bound chromatin underwent 3–4 washes and subsequent elution, decrosslinking, and purification steps. Diluted input and IPed DNA samples were subjected to quantitative real-time PCR using an Applied Biosystems Real-Time PCR System. Data normalization to input DNA levels and calculations were performed as previously described (39,44). The qPCR experimental setup and data analysis were conducted under the MiQE guidelines (43), and the checklist is provided in Supplementary Table S5. Further details regarding antibodies and primers can be found in Supplementary Tables S1 and S2, respectively.

### *IgH/c-Myc* translocation assay

Genomic DNA was isolated from CH12F3-2A cells transfected with control or gene-specific siRNA prior to CIT stimulation for 48 h. A standard phenol:chloroform extraction procedure was followed to isolate the DNA, which was used for PCR amplification of the *IgH/c-Myc* translocation junctions of the ch15 derivative (45,46). For the 1st PCR, 750 ng of DNA was used per PCR, and the PCR conditions were 94°C for 3 min, followed by denaturation at 94°C for 15 seconds, annealing at 62°C for 15 s and extension at 68°C for 7 min and 20 s. A total of 25 cycles were performed with a final extension at 68°C for 5 min. Nested PCR was performed using 1 μl of the first PCR product in a total volume of 20 μl, following the same PCR cycles with a final extension at 68°C for 7 min. The 2^nd^ PCR products were analyzed by agarose gel electrophoresis, transferred to a membrane, and subjected to Southern hybridization using a 5′DIG-labeled *c-Myc* probe. The entire assay procedure, including the translocation frequency calculation, was conducted mainly following the procedure described by Boboila *et al.* (47). Primer sequences *c-Myc* probe are described in Supplementary Table S2.

### Analysis of CSR junctions

Genomic DNA was isolated from siControl-, siBrd2- and siNipbl-treated CH12F3-2A cells stimulated with CIT for 48 h. First, the switched cells (IgA+) were stained with PE-anti-IgA and captured by anti-PE magnetic particle-DM capture (BD Biosciences), followed by genomic DNA isolation. The Sμ-Sα junction amplification was performed in two-step PCR using Pyrobest DNA Polymerase (TaKaRa). Briefly, 150 ng of isolated DNA was subjected to a 1^st^ round of PCR (2 min at 95°C followed by denaturation at 98°C for 10 s and annealing and extension at 68°C for 7 min for 22 cycles). A second round of PCR was performed using the purified 1^st^ PCR product and following a similar cycling condition with annealing at 68°C for 1 min and 20 s for a total of 24 cycles. Primers used for PCR amplification of the Sμ–Sα junctions are shown in Supplementary Table S2. The nested PCR products derived from the Sμ-Sα junctions were subjected to agarose gel electrophoresis, and fragments ranging from approximately 500–1000 bp were gel extracted and cloned. Several colonies from independent experiments were picked for plasmid preparation, followed by Sanger sequencing. The sequence of each clone was individually analyzed by alignment with the reference sequences (Sμ: AH005309 and Sα: D11468.1) using pairwise nBLAST at NCBI. The percentage of C-NHEJ or A-EJ was calculated from the total number of unique junctions analyzed and described in the figure legend. The two-tailed Fisher's exact test was performed for statistical significance determination. An organized view of the CSR junction sequences, adapted from Sundaravinayagam *et al.* (48), is shown in Supplementary Table S3.

### Immunoprecipitation/coimmunoprecipitation

To conduct immunoprecipitation (IP), we extracted either whole-cell protein by lysing the cells with modified RIPA buffer or nuclear extract using the NE-PER kit from Thermo Fisher. The cleared cell extract was incubated with Dynabeads® Protein G precoated with the antibody of interest. Depending on the requirements, ectopically expressed Flag- or EGFP-tagged proteins, anti-FLAG M2 agarose beads (Sigma) or GFP-Trap agarose beads (ChromoTek) were used. The IPed beads were washed 3–5 times at 4°C with modified RIPA buffer or with Pierce IP lysis buffer (Thermo Fisher) supplemented with an EDTA-free protease inhibitor cocktail and a phosphatase inhibitor mix. The final samples were prepared in 1x SDS sample buffer containing β-mercaptoethanol and heated at 85°C for 10 min. For Flag- or GFP-tagged proteins, we performed immunoblotting of the IPed samples following a routine procedure as described previously. All relevant antibodies are listed in Supplementary Table S1.

### DSB assay by LMPCR

Ligation-mediated PCR of DNA double-stranded breaks was adopted from previously described methods (49,50). Briefly, genomic DNA was isolated from CH12F3-2A cells stimulated for IgA switching for 24 h. Live cells isolated by Percoll density gradient centrifugation were embedded in low-melt temperature agarose plugs. Plugs were carefully processed and incubated with a ligation mixture containing a double-stranded unidirectional oligo-linker. Ligation was continued overnight at 16°C and terminated by heating at 70°C for 10 min. Genomic DNA with incorporated linkers at the DSBs was extracted and subjected to PCR amplification of the break points by a 5′ Sμ-specific primer and a linker-specific primer. PCR was performed using threefold dilutions of the linker ligated DNA and KOD FX *Neo* polymerase (Toyobo). Using a DIG-labeled Sμ probe, Southern blot analysis of the PCR products was carried out to confirm the amplification of Sμ-specific DSBs. A region from the *Gapdh* gene locus was selected for PCR to monitor the input genomic DNA amount. The primers and probe sequence are listed in Supplementary Table S2.

### NHEJ reporter assay

The NHEJ artificial reporter construct and the cell line carrying the reporter construct (H1299dA3-1) were previously described (51). The H1299dA3-1 cell line was a gift from T. Kohno at the National Cancer Center Research Institute, Tokyo. siRNA transfection into H1299dA3-1 cells and subsequent analyses were performed as reported earlier (52). Briefly, the *I-Sce*I-expressing plasmid (pCBASce) was co-transfected with siBrd2 or siControl into the reporter cell lines by FuGENE6 Transfection Reagent (Roche). After 48 hours, transfected cells were harvested for FACS analysis as well as for genomic DNA isolation for PCR. The details of the PCR conditions and PCR primers (Supplementary Table S2) were described by Ogiwara et al (51).

### Chromosome conformation capture (3C) assay

The 3C assay in CH12F3-2A cells was essentially performed as previously described (53,54). Following gene knockdown and CSR stimulation, control and siRNA transfected cells were crosslinked with 1% formaldehyde for 5 min at room temperature and quenched with 0.125 M glycine for 5 min. The cells were subsequently lysed in ChIP-IT Express Kit (Active Motif) lysis buffer for further processing. The protein-DNA crosslinked chromatin was digested with *Hind* III restriction enzyme overnight at 37°C, deactivated, and subjected to ligation using T4 DNA ligase for 4.5 hours at 16°C. Proteinase K treatment was carried out overnight to reverse the crosslink, followed by DNA purification using standard phenol/chloroform extraction. The Eμ-3′RR and Sμ−Sα interactions were examined by PCR amplification of the DNA samples using specific primers designed at the end of the restriction sites for the respective loci. The *Gapdh* locus served as a negative control for 3C and to monitor input DNA; see Supplementary Table S2 for all primer information. PCR products were analyzed on a 2% agarose gel, stained with EtBr, and images were captured by Bio-Rad Gel Doc EZ imaging system. For a quantitative readout, the band intensity of the PCR products was measured using ImageJ software and normalized with Gapdh control from multiple experiments.

### R-loop/DRIP assay

The R-loop or DNA: RNA hybrid immunoprecipitation (DRIP) assay was adopted from published methods (55,56) and previously described in detail for CH12F3-2A cells (11). In brief, genomic DNA isolated from siRNA-transfected and CIT-stimulated CH12F3-2A cells was subjected to multiple restriction enzyme digestion, followed by RNase A treatment. After phenol: chloroform extraction, the purified DNA was divided to perform RNase H (–/+) treatment. Both treated and untreated samples were incubated overnight with DNA:RNA hybrid-specific S9.6 antibody at 4°C. The antibody-bound DNA:RNA hybrid fragments were pooled, washed, and eluted by Protein G Dynabeads. Eluted samples were treated with Proteinase K and purified for downstream analysis. Both groups of samples, RNase H treated and untreated, were subjected to qPCR. The data were normalized to the input DNA and presented as % input.

### CRISPR/Cas9-induced CSR and ROSA26-H3f3b translocation

For the CRISPR/Cas9-induced IgM to IgA switching assay in CH12F3-2A cells, we cloned Sμ- and Sα-targeting gRNAs (Sμ-gRNA and Sα-gRNA) into the SpCas9 vector PX458 (Addgene). The gRNA oligonucleotide sequences have been previously described (57) and are listed in Supplementary Table S2. Oligonucleotides were appropriately designed, annealed, and cloned into the *Bbs*I/*Bbs*I sites located downstream of the U6 promoter in the vector. The two constructs, Sμ-CRISPR/Cas9 and Sα-CRISPR/Cas9, were cotransfected in a 1:1 ratio into 1 × 10^6^ CH12F3-2A cells by electroporation using Amaxa 96-well Nucleofector SF solution (program # CM-150). After 24 and 48 h of transfection, CSR was monitored by surface IgA staining and FACS analysis.

**Figure 2.**
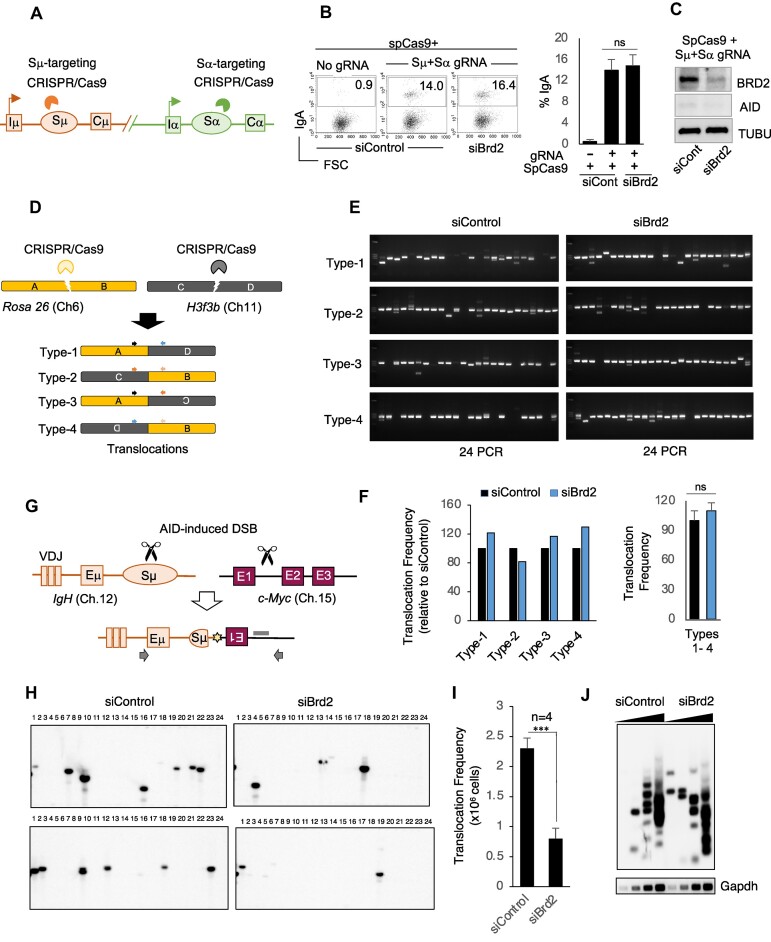
BRD2 depletion decreased AID-induced DNA aberrations but not DNA breaks. (A–C) *Brd2* knockdown did not perturb CRISPR/Cas9-induced CSR. (**A**) Schematic of Sμ- and Sα-targeting CRISPR/Cas9 to induce IgM to IgA switching. (**B**) FACS analysis of IgA switching in CH12F3-2A cells cotransfected with the indicated siRNA and SpCas9 plasmid harboring S region-targeting gRNAs. After transfection, cells were cultured for 24–48 h without CIT stimulation. The percentage of IgA-expressing cells is shown within each FACS plot, and the bar plots are a compilation of three independent experiments. (**C**) Representative Western blot of BRD2, AID and loading control using whole cell extracts of CH12F3-2A cells cotransfected with the indicated siRNA and CRISPR/Cas9 construct. (D–F) *Brd2* knockdown did not affect CRISPR/Cas9-induced chromosomal translocation in CH12F3-2A cells. (**D**) Schematic of CRISPR/Cas9-mediated DSB at *Rosa26* (Ch6) and *H3f3b* (Ch11) to induce *Rosa26/H3f3b* translocations. Colored arrowheads indicate PCR primers designed to detect four types of reciprocal translocations. (**E**) EtBr-stained agarose gel images of PCR-amplified *Rosa26/H3f3b* translocation junctions. Genomic DNA for PCR was isolated from CH12F3-2A cells co-transfected with the indicated siControl/siBrd2 and CRISPR/Cas9 plasmids. Three independent experiments showed a similar profile. (**F**) Summarized view of the relative frequency of four possible *Rosa26/H3f3b* reciprocal translocations, followed by a combined view. (G–I) *Brd2* knockdown decreased AID-induced *IgH*/c-*Myc* translocation in CH12F3-2A cells. (**G**) Schematic illustration of *IgH*/c-*Myc* translocation and detection strategy. The black arrows denote the PCR primer positions to amplify the recombined chromosomes. The gray bar denotes the position of the c-*Myc*-specific probe used for Southern hybridization. (**H**) Representative Southern blots of the *IgH*/c-*Myc* translocation assay of the indicated samples. (**I**) *IgH*/c-*Myc* translocation frequency calculated from four independent experiments. (**J**) *Brd2* knockdown did not affect AID-induced DSBs during CSR. Representative Southern blot of LM-PCR assay showing Sμ DSBs in CSR-activated CH12F3-2A cells transfected with siControl or siBrd2. Nested PCR for DSB detection was performed with 3-fold serial dilution (triangles) of the isolated genomic DNA. *Gapdh* PCR was performed as an internal control. (F and I) The values represent the mean ± SD (*n* = 3 or 4 experiments). Statistical analysis was performed using Student's *t*-test (***P ≤ 0.001; ns, not significant).

For the CRISPR/Cas9-induced chromosomal translocation assay in CH12F3-2A cells, we introduced DSBs in the *RSOA26* and *H3f3b* loci using a SpCas9 plasmid harboring gRNAs specific to each locus (Addgene). CH12F3-2A cells (1 × 10^6^ cells) were electroporated with either siControl or siBrd2 along with 2 μg of Cas9-gRNA (*Rosa26:H3f3b*) plasmid using an Amaxa 96-well system. Following electroporation, the cell suspension was diluted appropriately to distribute in a 96-well plate with a 1 × 10^4^ cells/well density. After 72 h of culture, the plate was centrifuged to collect cells from each well (∼1 × 10^5^ cells). We used Extraction Buffer-1 (40 μl) and Buffer-2 (4.5 μl) from the Takara Guide-it Mutation detection kit to lyse the cell pellet per well. Cell extracts from each well were subjected to nested PCR in a batch. In the 1st PCR, 5 μl of clarified cell lysate was used in a 50 μl reaction, followed by 2^nd^ PCR using 1 μl of 1st PCR in a 20 μl reaction mix. Final PCR products, 24 samples as a batch, were examined by running into 2% agarose gel and stained with EtBr. The PCR primers and PCR cycles related to the RSOA26 and H3f3b translocation assays were described previously (58,59). The *Rosa26:H3f3b* rearrangement, in all possible orientations (Figure 2D), was detected with high efficiency after 72 h of transfection of CRISPR/Cas9 in CH12F3-2A cells.

### DSB-end resection assay by qPCR

To evaluate the extent of resection adjacent to DSB at Sμ and Sα DSBs, qPCR was performed, following the method described previously (60,61). The extracted genomic DNA samples from siRNA transfected and CIT-stimulated CH12F3-2A cells were subjected to *Pvu*II/*Alu*I restriction endonuclease digestion using TaKaRa enzymes and buffer at 37°C for 4 h, in parallel with parallel mock-digestion. The resulting digested and mock-digested DNA samples were purified using phenol:chloroform extraction and ethanol precipitation. For each sample, 20 ng of equivalent DNA templates were used in a 20 μl qPCR reaction, containing 10 μl of 2× PowerUp SYBR Green Master Mix (Thermo Fisher) and 500 nM of each primer. The % ssDNA generated by DSB end resection at selected sites was calculated by subtracting the cycle threshold (Ct) value of the mock-digested sample from that of the digested sample, and then using the following formula: ssDNA% = 1/[2^(ΔCt-1)^ + 0.5] × 100 (61). The mean of at least three independent experiments was calculated and displayed as data, with SD values indicated by error bars. The qPCR primers are previously described (60) and listed in Supplementary Table S2.

## Results

### BRD2 promotes CSR induced by AID but not CRISPR/Cas9

Our previous study showed that the knockdown of Brd4 or treatment with JQ1, an inhibitor of the BET family of BRD proteins, impaired CSR (12). A loss-of-CSR function siRNA screen for the remaining BET members later identified Brd2 as another BET protein required for CSR (Supplementary Figure S1). We investigated the function of BRD2 in CSR using a mouse B-cell line (CH12F3-2A) that undergoes IgM to IgA isotype switching with high efficiency in response to CIT (CD40 ligand, IL-4, and TGF-b) stimulation (40). Knockdown of *Brd2* by siRNA showed a significant impairment of IgA class switching compared to the corresponding control, which correlated well with the knockdown efficiency (Figure 1A and B). We confirmed that the CSR impairment was not due to the transcriptional perturbation of the switch germline transcripts (μGLT and αGLT) or AID (Figure 1B and C). The result was further supported by the profile of the total RNA Poll (RNAPII) and the phosphorylated forms (S2P and S5P) of the elongating RNAPII across the S regions (Supplementary Figure S2). Similarly, depletion of *Brd2* in primary B cells also impaired IgG1 switching in response to LPS and IL4 stimulation without affecting GLT or AID transcription (Figure 1D–F).

To confirm the specificity of the knockdown, we expressed siRNA-resistant mouse *Brd2* with a Myc-Flag (MF) epitope tag at the C-terminus (Supplementary Figure S3). As expected, the expression of WT BRD2 from the *Brd2*^R^-MF construct fully restored siBrd2-mediated CSR inhibition in CH12F3-2A cells. However, the BRD2 mutants with deletion of the BD domains or the C-terminal BET domain could not completely rescue CSR, suggesting that both the BD and BET domains of BRD2 are critical for efficient CSR (Figure 1G–I).

Next, we asked whether BRD2 is similarly required for AID-independent CSR, such as by *CRISPR/Cas9*-induced CSR in CH12F3-2A cells (57). In this system, gRNA-guided Cas9 introduces site-specific DSBs at the donor and acceptor S regions and thus is independent of AID expression and GLT (Figure 2A). Transfection of Cas9 along with Sμ- and Sα-targeting gRNAs resulted in robust IgA switching in siControl-treated cells without CIT stimulation (Figure 2B). To our surprise, *Brd2* knockdown barely inhibited Cas9-induced CSR (Figure 2B and C), suggesting that BRD2 is specifically involved in AID-induced CSR in B cells under physiological conditions.

### BRD2 depletion restricts aberrant genomic recombination induced by AID

To investigate the role of BRD2 in genomic instability, we performed chromosomal translocation induced by CRISPR/Cas9- or AID-mediated DSBs in CH12F3-2A cells. We cotransfected Cas9 with gRNAs targeting the *Rosa26* and *H3f3b* loci into CH12F3-2A cells to induce chromosomal translocations between Rosa26 and H3f3b (58). We examined all possible combinations of reciprocal translocations as illustrated (Figure 2D). Surprisingly, we did not observe any significant difference in the frequency of *Rosa26/H3f3b* translocations between siControl- and siBrd2-treated cells (Figure 2E and F), indicating that BRD2 depletion does not affect CRISPR/Cas9-induced genomic instability.

Next, we conducted an *IgH/c-Myc* translocation assay (Figure 2G) in CH12F3-2A cells. During CSR, AID can induce DSBs at non-*IgH* loci, including the c-Myc locus, which is frequently translocated with the *IgH* locus in Burkitt lymphoma (46). Strikingly, BRD2 depletion reduced the frequency of AID-induced *IgH/c-Myc* translocations in CH12F3-2A cells, confirming its requirement in the AID-induced genomic instability pathway (Figure 2H, I). Therefore, BRD2 affects not only CSR but also the genomic aberrations originating from CSR-associated collateral DNA damage.

**Figure 3.**
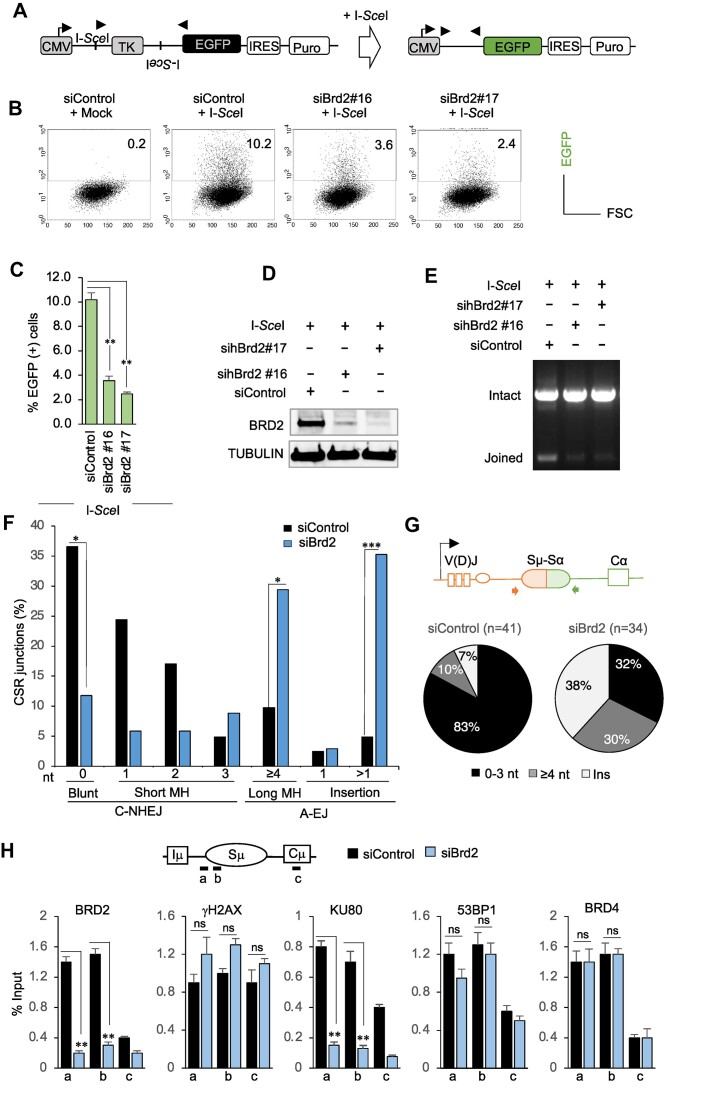
BRD2 promotes C-NHEJ-mediated joining of DSBs induced by *I-Sce*I or AID. (A–E) Knockdown of *BRD2* impaired repair of the *I-Sce*I cleaved DSB ends. (**A**) Schematic of the EGFP-reporter construct before and after *I-Sce*I cleavage and C-NHEJ. The EGFP shown in green is expressed after the thymidine kinase (TK) gene disappears following C-NHEJ. (**B**) FACS profile showing EGFP-positive cells after 24 h of cotransfection of the *I-Sce*I plasmid and the indicated siRNAs into cells with the integrated EGFP-reporter construct. (**C**) Compilation of %EGFP-positive cells detected by FACS analysis upon *BRD2* depletion from three independent experiments. (**D**) Western blot analysis showing the *BRD2* knockdown efficiency in the samples. (**E**) Images of EtBr-stained agarose gel show a representative result of genomic DNA analysis from indicated transfectants. Black arrowheads shown in A are the primers used for PCR. (F, G) Knockdown of *Brd2* decreased C-NHEJ-mediated repair of the CSR junctions. (**F**) Comparison of Sμ–Sα junctions between siControl- and siBrd2-transfected CH12F3-2A cells stimulated with CIT for 48 h. The percentage of switch junctions with the indicated length of microhomology (MH) or insertions was calculated and grouped under C-NHEJ or A-EJ (nt; nucleotide). Unique junctions were analyzed and collected from two independent experiments. Statistical significance was calculated with a two-tailed Fisher's exact test (*P ≤ 0.05, **P ≤ 0.01, ***P ≤ 0.001). (**G**) Pie charts comparing the distribution of Sμ–Sα junctions with short (0–3 nt) and long (≥4 nt) microhomology and total nucleotide insertion in siControl- and siBrd2-treated cells. Schematic representation of the recombined Sμ–Sα on the top shows the PCR primers' positions for junction analysis. (**H**) *Brd2* knockdown perturbs the occupancy of DDR and NHEJ factors in the S region. Top: An illustration of the Sμ region showing the positions of PCR amplicons of the ChIP−qPCR assay. Bottom: ChIP−qPCR analysis of BRD2, γH2AX, KU80, 53BP1 and BRD4 in CH12F3-2A cells transfected with siControl or siBrd2 and stimulated with CIT for 24 h. DNA input signals were used to normalize the ChIP data. (C and H) The data shows the mean ± SD (*n* = 3). Statistical analysis was performed using Student's *t*-test (*P ≤ 0.05; **P ≤ 0.01; ***P ≤ 0.001; ns; non-significant).

To examine whether AID-induced DSBs in the S region are perturbed, we performed a DSB assay by LMPCR, which allows the detection of S region-specific DNA breakpoints by PCR amplification (50). BRD2-depleted CH12F3-2A cells showed an LMPCR signal similar to that observed in the control (Figure 2J). Similarly, the DDR marker γH2AX was also elevated at the recombining S regions in response to CSR activation in both control and BRD2-depleted cells (Figure 3H). The results from both DSB assays were comparable between siControl and siBrd2, indicating a crucial role of BRD2 in repairing DNA breaks caused by AID. It also confirms that the reduced frequency of *IgH/c-Myc* translocation was not due to decreased AID-induced DSBs upon *Brd2* knockdown.

### BRD2 promotes non-homologous end joining but not homologous recombination

To investigate the impact of BRD2 depletion on DNA repair during CSR, which primarily relies on C-NHEJ, we initially examined its effect using a well-established C-NHEJ reporter assay (Figure 3A). In this assay, a reporter construct containing two *I-Sce*I sites in opposite orientations was integrated into the genome of the H1299dA3-1 human lung carcinoma cell line (51). Upon transfection of the *I-Sce*I endonuclease, cleavage of these sites occurs, and subsequent C-NHEJ repair between the incompatible DSB ends removes the intervening HSV-TK-ORF sequence, leading to the expression of EGFP. Therefore, quantifying EGFP-positive cells is a reliable measure of C-NHEJ efficiency.

We transfected control and two siRNAs targeting BRD2 into the reporter cell line, along with the *I-Sce*I plasmid, and subsequently monitored the expression of EGFP (Figure 3B and C). Depletion of *BRD2* by both siRNAs resulted in a significant decrease in cells expressing EGFP compared to the positive control. Notably, siRNA#17, which exhibited the most pronounced effect on *BRD2* knockdown, also demonstrated the lowest number of EGFP-expressing cells (Figure 3C and D), indicating the highest impairment of the C-NHEJ-mediated repair of *I-Sce*I-induced double-strand breaks (DSBs). These findings were further supported by the analysis of genomic DNA from the transfected cells (Figure 3E). Specifically, the expected PCR product resulting from the repaired junction was observed in the positive control. In contrast, its detection was significantly reduced in BRD2-depleted samples, corresponding to a 3–4-fold reduction in EGFP-positive cells. Analysis of the repaired junctions derived from the I-*Sce*I-induced DSBs showed an altered junction pattern, including elevated microhomology (MH) and insertions, typical of AEJ upon BRD2 knockdown, confirming the C-NHEJ defect (Supplementary Figure S4A–C).

We also investigated the role of BRD2 in DNA repair through homologous recombination (HR) using the widely-used DR-GFP reporter system (62). The HR reporter construct has two tandem GFP genes with different mutations—the upstream SceGFP has an I-*Sce*I recognition site and two stop codons, while the downstream internal GFP (iGFP) is truncated at both ends. DSBs were introduced in SceGFP cassette using I-*Sce*I expression plasmids, triggering DNA repair by gene conversion through homology-directed repair, using the downstream iGFP as a donor template, thus resulting in GFP expression (Supplementary Figure S5A). As expected, introducing I-*Sce*I-expression plasmids into CH12F3-2A cells with stably integrated DR-GFP robustly induced GFP expression (Supplementary Figure S5B). However, the GFP-positive population was not negatively affected but slightly elevated in *Brd2* knockdown (Supplementary Figure S5C). This suggests that while BRD2 is dispensable for HR, its absence can promote homology-mediated DNA repair. Such a phenomenon can help reduce AID-induced genomic instability like *IgH/c-Myc* translocation by promoting an error-free repair pathway (63).

### BRD2 depletion impaired C-NHEJ-mediated repair of CSR junctions

Considering that *Brd2* knockdown in B cells did not exhibit any defects in DSB formation, we investigated whether the absence of BRD led to C-NHEJ defects. Impairment in C-NHEJ often results in residual switching via the A-EJ pathway, which relies on MH-mediated repair. To explore this, we analyzed the Sμ-Sα junction patterns in control and BRD2-depleted CH12F3-2A cells induced for CSR. In C-NHEJ, direct joining or blunt ligation (0 nt) is the dominant feature, although occasional junctions may display short MHs of up to three nucleotides (≤3 nt). For differentiation between C-NHEJ and A-EJ, we considered ≤3 nt MH as indicative of C-NHEJ, while ≥4 nt MH and any nucleotide insertion were attributed to A-EJ (Figure 3F).

Compared to control cells, BRD2-depleted cells exhibited a significant decrease in blunt-end ligation (≤3 nt) and a simultaneous increase in longer MH (≥4 nt) at Sμ–Sα recombination junctions (Figure 3F, G and Supplementary Table S3). Additionally, a small percentage of junctions in BRD2-depleted cells displayed insertions of one or two base pairs, as well as insertions of longer fragments (>30 bp). Notably, these longer fragments were predominantly derived from different genomic locations, reminiscent of the phenomenon known as ectopic capture of chromosomal sequences (ECSs) (64). Overall, a wide range of altered junctional features was observed in BRD2-depleted cells, including insertions, MH, and ECSs between donor and acceptor S regions. The result is also consistent with the analyses of the repaired junctions of I-*Sce*I-induced DSBs in BRD2-depleted NHEJ reporter cells ( Supplementary Figure S4A–C). These findings strongly support the role of BRD2 in suppressing spurious joining and promoting C-NHEJ for efficient CSR.

To investigate the potential dysfunction of C-NHEJ-mediated end joining, we examined the recruitment of DDR and C-NHEJ factors in the S region of CH12F3-2A cells treated with either siControl or siBrd2, followed by CSR stimulation with CIT. Although knockdown of *Brd2* reduced BRD2 occupancy in the S regions, the enrichment of γH2AX was comparable between the control and knockdown samples (Figure 3H). This result aligned with the DSB assay by LMPCR, which indicated that *Brd2* depletion does not perturb AID-induced DSB formation in the S region during CSR (Figure 2J). Consistent with the C-NHEJ defect observed in *Brd2* knockdown cells, we observed decreased KU80 occupancy, indicating impaired C-NHEJ complex formation. As expected, I-*Sce*I-induced DSB sites in the NHEJ reporter locus similarly reduced KU80 recruitment despite elevated γH2AX deposition upon BRD2 depletion (Supplementary Figure S6B). Unaltered 53BP1 and BRD4 occupancy in the S region (Figure 3H) also suggests that unlike BRD4 (12), BRD2 does not promote 53BP1 recruitment in B cells.

### BRD2 does not regulate L3MBTL or ZYMND8 occupancy in the S region

Previous studies have reported the enrichment of BRD2 on acetylated chromatin near site-specific zinc finger nuclease-induced DSBs in 293T cells (44). It was shown that BRD2 is required to remove the 53BP1 repressor L3MBTL1 by the VCP/p97 ATPase (65), which facilitated the recruitment of 53BP1 to the DSB site. Furthermore, BRD2 promoted DNA repair by facilitating the recruitment of ZMYND8 and protecting acetylated chromatin regions flanking the DSBs. Therefore, we aimed to investigate whether BRD2 functions similarly by regulating L3MBTL1/ZYMND8 recruitment during CSR.

**Figure 4.**
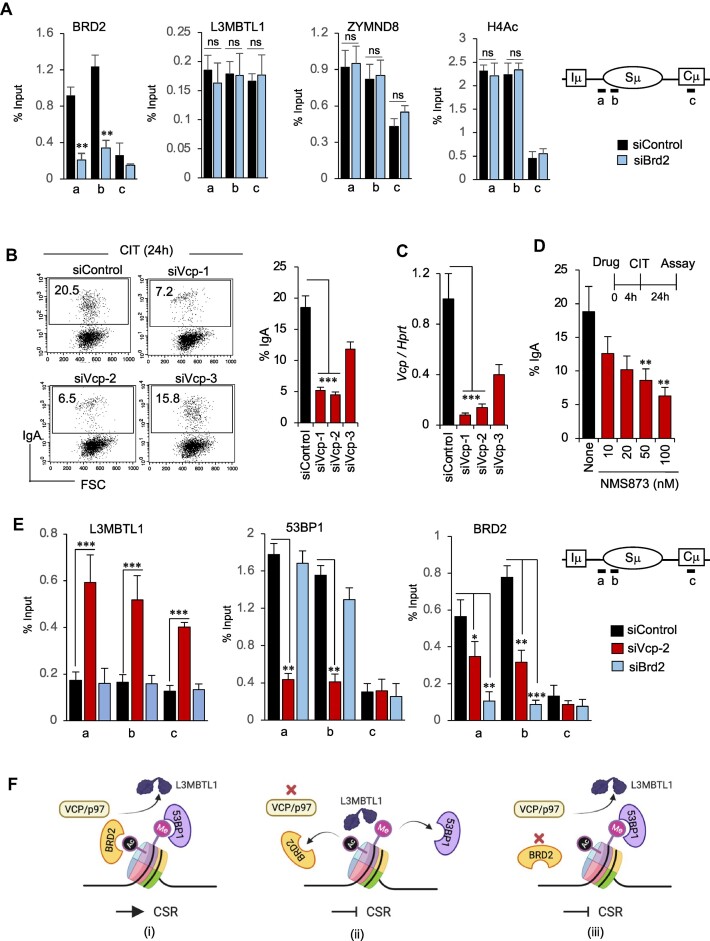
VCP/p97 ATPase regulates 53BP1 and BRD2 occupancy in the S region. (**A**) ChIP−qPCR for BRD2, L3MBTL1, ZYMND8 and H4Ac in the Sμ region of the *IgH* locus in CH12F3-2A cells transfected with siControl or siBrd2. siRNA transfection and CSR activation by CIT were performed as depicted in the upper panel of Figure 1A. The positions of qPCR amplicons (a, b and c) in the Sμ are indicated in a diagram on the right. Enrichments are calculated relative to the ChIP input from three experiments. (**B**) Effect of VCP/p97 ATPase knockdown or inhibition of the catalytic activity on CSR. Representative FACS plots showing CSR to IgA in activated CH12F32A cells transfected with siControl or siRNA Vcp/p97 ATPase. The bar graphs next to the FACS profile summarize the CSR efficiency from three independent experiments. (**C**) Evaluation of Vcp/p97 knockdown by RT−qPCR of the total RNA isolated from the indicated siRNA-transfected CH12F3-2A cells. (**D**) Analysis of CSR to IgA in CH12F3-2A cells treated with increasing concentrations of NMS873, a pharmacological inhibitor of VCP/p97 ATPase. The data are from two independent experiments. (**E**) Knockdown of *Vcp/p97* but not *Brd2* affected L3MBTL1 and 53BP1 occupancy in the Sμ region. ChIP−qPCR for L3MBTL1, 53BP1, and BRD2 in the Sμ region of CIT-stimulated CH12F3-2A cells. The transfected siRNAs and the position of the qPCR amplicons in the Sμ region are shown on the right. The data represent three independent experiments. (**F**) Illustrated overview of the independent requirement of VCP/p97 and BRD2 in CSR. (i) In wild-type, when CSR is optimal, Vcp/p97 removes the L3MBTL1 repressor at the DSB, allowing normal 53BP1 recruitment. Vcp/p97 also promotes BRD2 recruitment in the S region. (ii) In the absence of Vcp/p97, CSR was impaired due to 53BP1 and BRD2 recruitment defects. Failure of L3MBTL1 removal from H4K20me1/2 interfered with 53BP1 binding. (iii) In the absence of BRD2, 53BP1 occupancy in the *IgH* locus DSB was normal, as both Vcp/p97 and BRD4 (Figure 3H) were available. However, CSR inhibition points toward a defect other than the L3MBTL1/53BP1 pathway in Brd2 deficiency. (A–E) The data is presented as mean ± SD (*n* = 3 or 2 experiments) Statistical analysis was performed using Student's *t*-test (*P ≤ 0.05; **P ≤ 0.01; ***P ≤ 0.001; ns, non-significant).

Contrary to our expectation, *Brd2* depletion did not reveal any changes in L3MBTL1 or ZMYND8 occupancy in the S region (Figure 4A), although γH2AX was elevated due to unperturbed AID-induced DSBs (Figure 3H). There was also no change in histone acetylation (H4Ac) status in the locus despite a significant reduction of BRD2. To further verify the regulatory pathway, we knocked down VCP/p97 or inhibited its ATPase activity by a pharmacological inhibitor, NMS873 (66). In both cases, we observed reduced CSR (Figure 4B-D), suggesting that VCP/p97 positively regulates CSR and is required for L3MBTL1 removal in B cells. The ChIP analysis confirmed that the depletion of VCP/p97 resulted in an increase in L3MBTL1 occupancy and a simultaneous decrease in 53BP1 occupancy in the S region (Figure 4E; first two panels). Interestingly, the occupancy of BRD2 was also reduced in the S region (Figure 4E; last panel), indicating that VCP/p97 also regulates the deposition of BRD2 during CSR. Therefore, VCP/p97 is a critical CSR regulator that impacts not only 53BP1 but also BRD2 deposition in the S region (Figure 4F).

Given that BRD2 depletion did not affect 53BP1, L3MBTL1, or ZMYND8 occupancy in the S region (Figures 3H and 4A), we conclude that BRD2-mediated DNA repair in CSR does not involve the L3MBTL1/53BP1 inverse regulatory cascade associated with Vcp/p97. We also did not observe any effect on S region H4Ac or ZMYND8 occupancy in *Brd2* knockdown (Figure 4A), which is also in line with the DNA repair-unrelated function of ZMYND8 in CSR (67).

### BRD2 functions together with NIPBL to promote S−S recombination

**Figure 5.**
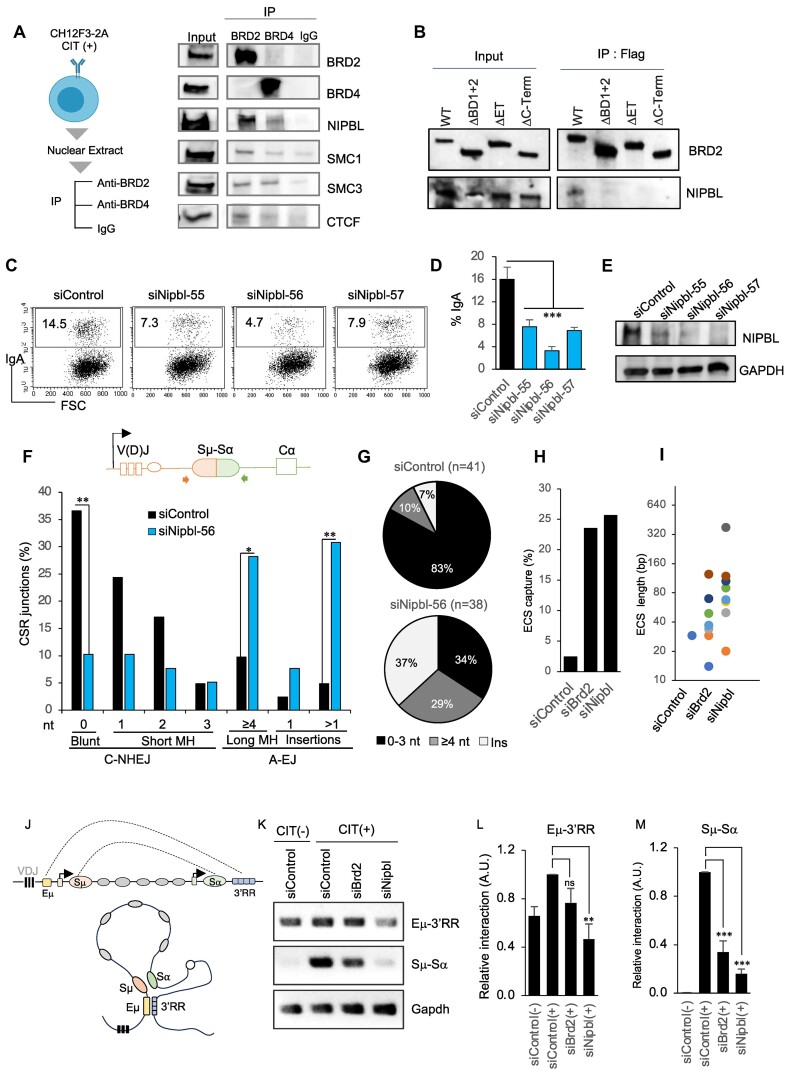
BRD2 and NIPBL interact and regulate S−S synapsis, A-EJ and ECS capture. (A, B) Interaction of BRD2 and NIPBL in CSR-activated B cells. (**A**) Left: Schematic of IP. Nuclear extracts from activated CH12F3-2A cells were immunoprecipitated using BRD2 and BRD4 antibodies or control rabbit IgG. Right: Western blot analyses of the IPed proteins with the indicated antibodies. (**B**) BRD2 interacts with NIPBL through the ET and BD domains. As illustrated in Figure 1G, EGFP- and MycFlag-tagged BRD2 constructs were expressed in 293T cells and immunoprecipitated using GFP-Trap agarose beads. IPed proteins were analyzed by Western blot for BRD2 and NIPBL, as indicated on the right in the respective panel. The WT and the BRD2 mutants used for the IP are indicated at the top. The data shown is representative of three independent experiments. (C–E) Impact of *Nipbl* knockdown on CSR to IgA. (**C**) FACS analysis of CH12F3-2A cells transfected with the indicated siRNAs and stimulated with CIT as in Figure 1A. The percentage of IgA (+) cells is indicated in the respective FACS plot. The data are representative of more than three experiments. (**D**) Summary of three independent *Nipbl* knockdown experiments in CH12F3-2A cells. (**E**) Representative immunoblot analysis of NIPBL and GAPDH as a loading control using whole cell extracts from the transfected CH12F3-2A cells as indicated. (F–I) Like *Brd2* deficiency, *Nipbl* loss elevates A-EJ and ECS capture at S−S junctions. The bar plots (**F**) and the pie charts (**G**) represent the Sμ-Sα recombination junction analysis. Genomic DNA was isolated from activated CH12F3-2A cells transfected with siControl or siNipbl. The experimental procedure and the analysis of CSR junctions were the same as for Figure 3F and G. The control data were taken from Figure 3F. Statistical significance was calculated with a two-tailed Fisher's exact test (*P ≤ 0.05, **P ≤ 0.01, ***P ≤ 0.001). (**H**) Knockdown of *Brd2* or *Nipbl* increases the ECS insertion frequency at the Sμ–Sα junctions. (**I**) Size distribution of the ECSs incorporated at the Sμ–Sα junctions; each dot represents an ECS insertion. (J–M) BRD2 or NIPBL depletion disrupts Sμ-Sα break-end synapse. (**J**) Schematic of the *IgH* locus depicting conformational S−S proximity during CSR. (**K**) EtBr-stained gel image of PCR products from the chromosome conformation capture (3C) assay using genomic DNA isolated from CH12F3-2A cells transfected with the indicated siRNAs. Sequencing of the 3C-PCR products confirmed the expected *Hind*III–*Hind*III ligation junction between Eμ-3′RR and Sμ-Sα and the Gapdh locus as a control. (**L**, **M**) The band intensities of the PCR products were quantified by ImageJ software. Each data point was normalized to the corresponding Gapdh control and plotted relative to CIT-stimulated (+) and siControl-treated samples. The data represents the average of four independent experiments ± SD; Statistical significance was measured by Student's *t*-test (**P ≤ 0.01; ***P ≤ 0.001; ns; non-significant).

We speculated that the mode of AID-induced DSB repair by BRD2 may opt different pathways distinct from 53BP1/L3MBTL1/ZYMND8 in 293T cells or BRD4/53BP1 in B-cells (12,44). Interestingly, during T lymphocyte (Th17) differentiation, BRD2 and BRD4 exert distinct functions through differential interactions with the cohesin complex (68). We performed immunoprecipitation (IP) of BRD2 and BRD4 to explore a similar possibility in activated B cells. We found that BRD2 and BRD4 indeed interact differently with cohesin complex proteins in CH12F3-2A cells undergoing CSR (Figure 5A). As observed in Th17 cells (68), NIPBL showed a preferential interaction with BRD2 over BRD4. SMC1 and CTCF also showed a higher affinity for BRD2 over BRD4, although the interaction was weaker than that of NIPBL-BRD2. To verify our findings, we conducted an additional experiment using WT and BRD2 mutants that failed to complement CSR (Figure 1G–I). Immunoprecipitation of WT BRD2, but not mutant BRD2, pulled down the cohesin loader NIPBL. Deletion of the ET domain or the C-terminus encompassing the ET domain in BRD2 completely abrogated the interaction between BRD2 and NIPBL (Figure 5B). The double BD domain deletion mutant also showed reduced interaction, suggesting that BRD2 interacts with NIPBL through its ET domain while bound to acetylated chromatin. This result is in line with the failure of CSR complementation by BRD2 mutants lacking the BD or ET domain (Figure 1G–I).

Next, we examined the effect of NIPBL loss on CSR and Sμ–Sα repair junctions in CH12F3-2A cells. We tested three different siRNAs targeting *Nipbl* and found that all of them effectively inhibited CSR and showed good knockdown efficiency (Figure 5C–E). However, we chose siRNA-56 for further study due to its particularly strong effect on CSR (Figure 5D). We confirmed that the transient depletion of *Nipbl* by siRNA has no adverse effect on switch germline transcription and RNAPII/ RNAPII S2P loading on the recombining S regions (Supplementary Figure S7B–D). Although sufficiently present in the recombining S regions, RNAPII S5P showed a slight decreasing trend in the NIPBL-depleted condition (Supplementary Figure S7E). Surprisingly, the CSR junctions from NIPBL-depleted cells showed a significantly reduced DNA repair by C-NHEJ with a concomitant elevation of MH- or A-EJ-mediated repair (Figure 5F and G), similar to the observations in *Brd2* depletion (Figure 3F and G). Our result is consistent with a previous report that NIPBL depletion decreases NHEJ efficiency during CSR (35). Notably, a bias toward A-EJ-mediated repair of the CSR junctions has also been reported in B lymphocytes from patients with CdLS who have heterozygous loss-of-function mutations in the *NIPBL* gene (34).

Furthermore, we observed a marked increase in the insertion frequency (>1 nt) in both *Brd2* and *Nipbl* knockdowns (Figure 5F and G). Upon closer examination, we found that the inserted fragments at the CSR junctions originated from distant parts of the genome and were longer (>30 nt), resembling the phenomenon of ECS capture (64). Distal DSB repair by ECS capture has been observed in cohesin complex deficiency and is thought to occur through MH-mediated template switching (64,69). We observed a similar ECS profile between BRD2 and NIPBL deficiencies, including a rare incident of capturing the same genomic region in both (Figure 5H, I).

Since the NIPBL/Cohesin complex is involved in the long-range genomic conformation and accurate repair of distal DSBs (69,70), we were curious to examine whether the Sμ-Sα synapse during CSR remains intact upon BRD2 and NIPBL loss. To monitor the *IgH* locus conformation (Figure 5J), we investigated the interaction between the Eμ enhancer and 3′RR super-enhancer (Eμ-3′RR) as well as the interaction between Sμ and Sα using a 3C assay (Figure 5K–M). The Eμ-3′RR interaction, constitutive and modestly enhanced upon CIT stimulation, was primarily affected by *Nipbl* knockdown and less *Brd2* KD (Figure 5K and L). Conversely, the Sμ-Sα interaction, which is typically elevated upon CSR stimulation [compare siControl (–) versus siControl (+)], was significantly decreased after *Brd2* or *Nipbl* knockdown (Figure 5K and M). This result indicates a defective break-end synapse without BRD2 and/or NIPBL, which may not support proper C-NHEJ repair complex formation. Combined with the Sμ–Sα junction data and the observed BRD2-NIPBL interaction, we conclude that BRD2 deficiency shares a common or overlapping DNA repair defect with NIPBL deficiency.

### The stability of BRD2-NIPBL on the S-region is essential to elicit optimal DDR

**Figure 6.**
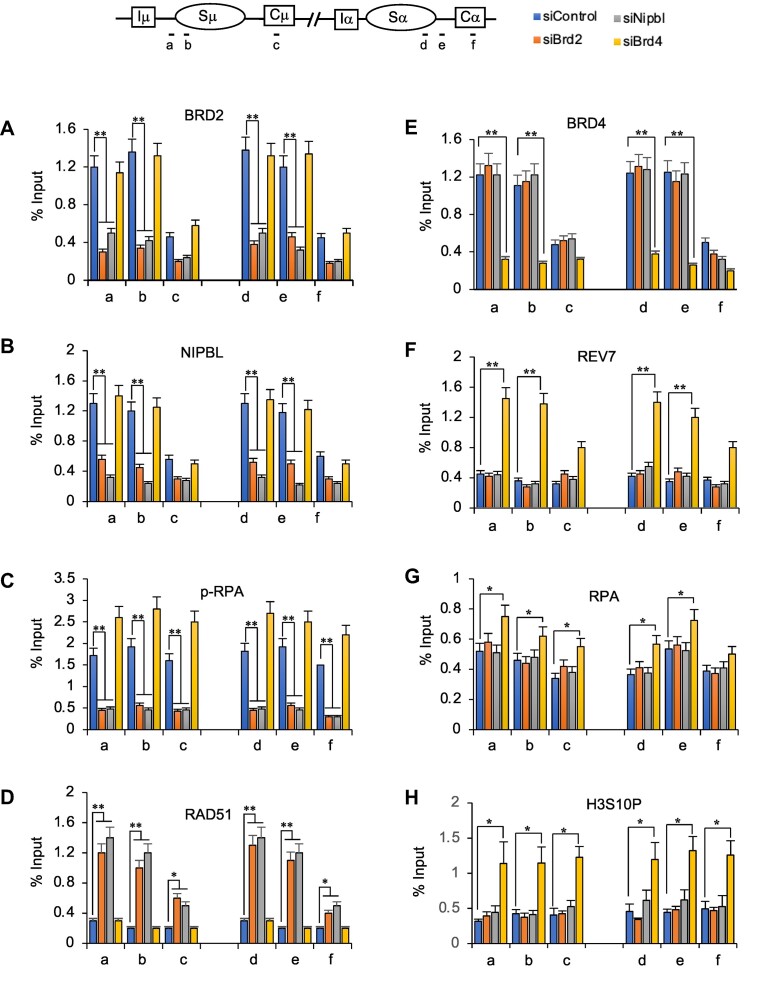
Brd2-Nipbl cooperation underlies the efficiency of CSR and C-NHEJ. (**A–D**) Depletion of BRD2 and NIPBL affects their and other DDR factor occupancies similarly on the recombining S region. ChIP−qPCR for BRD2, NIPBL, p-RPA and RAD51 occupancy showed responsiveness to *Brd2* and *Nipbl* knockdown but remained unaffected by *Brd4* knockdown. (**E–H**) ChIP−qPCR of BRD4, REV7, RPA, and R-loop formation (S9.6) in the recombining S region. Brd4 depletion increased REV7, RPA and R-loops, which remained comparable to the control upon BRD2 or NIPBL depletion. BRD4 depletion does not affect BRD2 or NIPBL occupancy (A, B). The siRNA transfection and CSR activation of CH12F3-2A cells by CIT was performed as stated in Figure 1A. The positions of qPCR amplicons (**a–f**) in the *IgH* Sμ and Sα regions are indicated in the upper panel. Enrichments are calculated relative to the ChIP input. (A-H) The data shows the mean ± SD. Statistical analysis was performed using Student's *t*-test (*P ≤ 0.05; **P ≤ 0.01).

The preferred interaction and functional similarity between BRD2 and NIPBL (Figure 5A) led us to investigate the effect of *Brd2* knockdown on NIPBL occupancy at the recombining S regions. ChIP analysis was performed for NIPBL and BRD2 on Sμ and Sα in CH12F3-2A cells depleted of NIPBL, BRD2 or BRD4 during CSR stimulation. (Figure 6A–H). Consistent with a previous report, NIPBL was detected in both Sμ and Sα in CSR-activated CH12F3-2A cells (70). Surprisingly, *Brd2* but not *Brd4* knockdown significantly reduced NIPBL loading in the S regions (Figure 6B; compare orange versus yellow bars). Similarly, the *Nipbl* knockdown also affected BRD2 but not BRD4 occupancy (Figure 6A and E; gray bars), which is consistent with the preferential interaction between BRD2 and NIPBL (Figure 5A). Deposition of REV7, which is a C-NHEJ-associated repair factor in the 53BP1/Shieldin complex, was elevated only upon *Brd4* knockdown (Figure 6F, yellow bars). Strikingly, phosphorylated RPA2 (p-RPA) was drastically reduced in both *Brd2* and *Nipbl* knockdown (Figure 6C; see orange and gray bars) despite total RPA2 (RPA) elevation in the S regions (Figure 6G). In contrast, p-RPA was dramatically increased along with total RPA in *Brd4* knockdown (Figure 6C; yellow bars), suggesting that the two events are parallel during CSR. When AID-induced DSB causes a high degree of DNA resection, RAD51 is extensively deposited at the *IgH* locus, analogous to the RPA (71,72). Strikingly, we also observed an increase in the deposition of RAD51 upon knockdown of *Brd2* and *Nipbl* but not *Brd4* (Figure 6D).

Although recent studies have indicated that R-loops may play a role in DNA repair (13,15,73), the S region-specific R-loops’ primary function is facilitating AID-induced DNA breaks. To determine the extent of R-loop formation in the S region, we used the S9.6 antibody to pull down DNA:RNA hybrid sensitive to RNase H-mediated degradation. Our results showed that knockdown of *Brd4*, but not *Brd2*, increased R-loop accumulation (Supplementary Figure S8). This finding also agrees with a recent report demonstrating that loss of BRD4 but not BRD2 leads to the accumulation of R-loops in cancer cells (74). H3S10P, a chromatin modification intimately associated with R-loop and DNA damage (75–77), has also been found to be upregulated in *Brd4* deficiency (Figure 6H), further emphasizing that BRD2 and BRD4 function distinctly in CSR. Taken together, we conclude that BRD2, but not BRD4, functions mainly in cooperation with NIPBL to promote C-NHEJ repair by suppressing A-EJ and ECSs. This phenomenon is likely caused by the activation of DSB end resection due to the loss of DSB protective factors such as KU80 and topological perturbation upon NIPBL loss (63,70,78). Impaired RPA phosphorylation may also play a role, as p-RPA is a crucial DDR that prevents excessive DNA resection by slowing down ‘resectosome’ formation on the chromatin template (79).

### Knockdown of Brd2 increases DNA end resection in the S region

To confirm that BRD2 deficiency impacts DNA end resection of S-region DSB, we conducted additional experiments to examine the recruitment of DNA end processing factor and the accumulation of single-stranded DNA (ssDNA) intermediates resulting from end-resection. Resection of DSB requires coordinated actions of various nucleases and helicases—CtIP initially stimulates the MRE11-mediated short resection, followed by further resection of the DSB by EXO1/DNA2 nucleases and BLM/WRN helicase (80,81). The 3′ ssDNA overhangs that undergo RPA and, subsequently, RAD51 coating promote homology-directed repair. To gain insights into the role of BRD2 in DNA end resection, we first examined the recruitment of CtIP, EXO1, and BLM in CSR-activated CH12F3-2A cells. ChIP analysis revealed that there was no marked accumulation of CtIP in the S region upon BRD2 depletion, but there was a substantial enrichment of EXO1 and BLM in the recombining S regions (Supplementary Figure S9A), indicative of a potential cause in elevated resection at the S regions DSBs.

To further provide more direct evidence of increased DNA end resection upon BRD2 depletion, we applied a quantitative approach to monitor ssDNA intermediates generated by the resection of Sμ and Sα DSBs (60,61). The principle of the assay and the position of qPCR amplicons (p1-p5) on *IgH* or Cd3e gene loci are shown (Supplementary Figures S9B and C, top). The Cd3e gene locus was utilized as a negative control locus, which does not undergo AID-induced DSB. As *Pvu*II has been chosen as the restriction endonuclease (RE), sequencing of our CH12F3-2A line confirmed that each PCR target region (p1–p5) harbors a PvuII site. Based on this assay, the PvuII-resistant PCR product indicates the extent of ssDNA, which was calculated by comparing it with the mock digested DNA from the same sample. In siControl-treated cells, PvuII-resistant ssDNA was 0.5–3% with an increasing trend toward the core Sμ (p3). *Brd2* knockdown increases PvuII-resistant ssDNA 2–3fold over siControl treated sample in both Sμ and Sα (Supplementary Figure S9C). Downstream of p3, we have selected a region that harbors three *Alu*I sites; when we measured *Alu*I-resistant ssDNA at that region, it produced a very similar result (not shown), further confirming the increase of ssDNA in the S region after *Brd2* knockdown. Only a negligible signal was detected from the negative control locus.

To validate the assay, we knocked down Shieldin component *Shld2*, which is known to function with 53BP1 in DSB end protection. In Shieldin deficient cells, NHEJ is suppressed with concomitant elevation of A-EJ, DNA end resection, and ssDNA accumulation (82). Strikingly, under this condition, we observed a similar trend of PvuII-resistant ssDNA profile as observed in BRD2 deficiency (Supplementary Figure S9D). Interestingly, when we simultaneously knocked down *Brd2* and *Shld2*, the PvuII-resistant ssDNA level was further elevated, which may suggest their independent role in DSB-end resection. Indeed, this hypothesis is supported by the observation that BRD2 depletion does not affect 53BP1 accumulation in the S-region (Figure 3H). Taken together, we conclude that BRD2 functions as an NHEJ factor, and its absence, similar to that of a known DSB-end protecting factor, induces extensive ssDNA resection.

## Discussion

Several BRD family genes are expressed in both CH12F3-2A cells and the germinal center B-cells, as shown in Supplementary Figure S1A,C,and D. With a particular focus on BET family chromatin proteins, we demonstrate that in addition to BRD4 (12), BRD2 plays a crucial role in CSR, while BRD3 is dispensable (Supplementary Figure S1B). To decipher the role of BRD2 in CSR, we first determined whether it is involved in the S region DNA cleavage formation or at the subsequent S-S joining phase — the two well-documented phases involved in CSR. AID-induced DNA cleavage requires switch GLTs responsible for R-loop and G4 structure formation, leading to the generation of sufficient ssDNA substrate for breakage. The GLTs and DSBs in the S region remained unchanged in *Brd2* knockdown, consistent with the unperturbed R-loop and γH2AX accumulation in the S region (Figures 2J and 3H). BRD2 is dispensable for DSB initiation while critical for CSR, suggesting that BRD2 is involved in the joining phase of CSR, which predominantly occurs through the NHEJ pathway.

As predicted, CSR junctions from BRD2-depleted B cells exhibited a sharp decrease in direct joining along with an increase in A-EJ or MH-mediated repair (Figure 3F and G), a typical feature of C-NHEJ deficiency (83,84). Consistently, the C-NHEJ-dependent repair of I-*Sce*I-cleaved DSBs in the C-NHEJ reporter system was significantly compromised in BRD2 deficiency (Figure 3B-E). Consequently, under this condition, both the S regions and the NHE-J reporter locus, exhibited impaired KU80 recruitment in response to AID and I-*Sce*I-induced DSB, respectively (Supplementary Figure S6B). BRD2 deficiency led to more extensive resection of free DNA ends and an increased level of EXO1 and BLM — a common occurrence that impedes C-NHEJ (Supplementary Figure S6C). Although we had initially anticipated an increase in the ssDNA binding protein RPA in the resected region upon BRD2 depletion, its accumulation did not exceed that of the control sample. Instead, we observed an increase in the occupancy of RAD51 under the same condition. As RAD51 subsequently displaces RPA from the resected DNA, we interpret this finding from the perspective of the inherently dynamic relationship between the two factors, whereby the increase in RPA might not be readily apparent or detected. Moreover, a more direct measurement of ssDNA resection in the absence of BRD2 in the S regions provided a comparable result with *Shld2* deficiency (Supplementary Figures S9C and D). This emphasizes a potential role for BRD2 in DSB protection but is distinct from the Shieldin/53BP1 pathway (83,84). The finding also agrees with the observation that the loss of BRD2 did not affect 53BP1 recruitment in the S region during CSR (Figure 3H).

We demonstrated that BRD2 binds to the S region chromatin and requires its BD1 and BD2 domains for CSR (Figure 1G-I), which recognize acetylated histones that elevate upon CSR induction (12,20). The ET domain of BRD2 interacted with NIPBL (Figure 5B) as reported previously in a yeast two-hybrid assay (31). Although the ET domain is present in BRD2 and BRD4, BRD2 predominantly interacted with NIPBL in CSR-activated B cells. Moreover, deleting the two BD domains also considerably reduced NIPBL pull-down with BRD2, suggesting that the BRD2-NIPBL interaction occurs in a chromatin environment involving ET and BD domains. Accordingly, we observed a failure of CSR complementation by BRD2 lacking the ET or BD domain. Furthermore, BRD2 depletion reduced NIPBL occupancy in the S region (Figure 6B), suggesting that BRD2 tethered on acetylated chromatin interacts with NIPBL and destabilizes it when knocked down. Therefore, we noted a functional overlap between BRD2 and NIPBL upon their depletion, including elevated A-EJ and ECS at the CSR junctions, S-S synapse, and impaired DDR (Figures 5 and 6).

The defective S-S synapse and the loss of NIBPL from the S regions in BRD2-depleted cells suggest an altered topological landscape in the *IgH* locus, which can hamper C-NHEJ during CSR. NIPBL loss could also explain why processes like CSR and AID-dependent translocations that involve chromatin extrusion are more affected than CRISPR-induced ones, pointing to a significant role of chromatin topology dynamics (70,85). Because, it is essential for the recombining S regions to come in proximity and stay in the correct configuration during C-NHEJ, which occurs through oriented S–S synapsis, allowing C-NHEJ/and or recombination machinery to act and execute successful deletional recombination (78). Loss of DSB end-protective factor can induce excessive DNA end-resection and elevate ECS capture during the repair of distal end DSBs, as observed in BRD2/NIPBL deficiency (64). The NIPBL/Cohesin complex has been reported to limit the ECS capture by constraining the mobility of the ssDNA and promoting accurate repair between distally located DSBs (69). The S–S synapse defects in BRD2/NIPBL deficiency may increase the resected free end mobility and interfere with the repair-recombination (11,86).

This study also revealed several distinct features between BRD2- and BRD4-mediated CSR, although deficiency of both causes C-NHEJ defect. A key difference is that BRD2 but not BRD4 depletion affects NIPBL deposition in the locus and thus does not overlap with NIPBL-associated functions such as *IgH* locus topology. BRD2 or NIPBL depletion showed no marked decrease in 53BP1 occupancy in the S region. In the case of CdLS-associated NIPBL mutations, there is also a contrasting report suggesting transient and longer 53BP1 foci formation (30,34). However, recruitment of NHEJ and CSR-associated repair factor MAD2L2/REV7 (87), which is downstream of 53BP1 and a component of Shieldin, remained unaffected in BRD2 and NIPBL deficiency. Conversely, there was a striking increase in MAD2L2/REV7 in the S-region after BRD4 depletion, which aligns with a recent report on CdLS-type BRD4 mutation (30). Moreover, BRD4, but not BRD2, depletion markedly elevated the R-loop in the S-region, which is associated with AID-induced DNA breaks (88,89). On the other hand, no S−S synapse defect or elevation of ECS capture at the S−S junction was evident only in BRD2 as well as in NIPBL deficiency. This emphasizes that although both BRD2 and BRD4 are required for CSR during NHEJ, their mode of action is nonoverlapping, and thus, the deficiency of one could not be complemented by the other.

It is currently unclear how various DNA-resection pathways intersect and/or are regulated during CSR, as failure to suppress the DNA resection pathways can compromise C-NHEJ. One of the striking common defects in BRD2 or NIPBL deficiency was a striking decrease in p-RPA (Figure 6C), which normally increases with RPA occupancy on the resected ssDNA (87,90). A recent study suggests that pRPA also can suppress homology-directed DNA repair condenstate formation, which is normally facilitated by non-phosphorylated RPA and ssDNA (91). Moreover, p-RPA has been reported to reduce the DNA end resection by EXO1/BLM on chromatin (79). Elevated levels of EXO1 and BLM were also observed in the S-region in BRD2 deficiency, concomitant with the reduction in p-RPA formation. We propose that the collaboration of BRD2 and NIPBL during CSR facilitates S-S synapsis and optimal DDR and C-NHEJ hub formation at the DSB site, safeguarding from excessive DNA end-resection. Further study in this direction will help unravel the exact mode of functional cooperation between NHEJ-promoting BET proteins and NIPBL, which are crucial for programmed DNA rearrangement and preserving genome stability.

## Supplementary Material

gkae204_Supplemental_File

## Data Availability

The data underlying this article are available in the article and in its online supplementary material.
