# Supplementary material for: BRD2 promotes antibody class switch recombination by facilitating DNA repair in collaboration with NIPBL
Source: Nucleic Acids Res. 2024 Apr 3;52(8):4422–39. doi: 10.1093/nar/gkae204 (PMC11077081; doi:10.1093/nar/gkae204)
Supplement: gkae204_Supplemental_File [file gkae204_supplemental_file.pdf]

BRD2 Promotes Antibody Class Switch Recombination by Facilitating DNA Repair in Collaboration with NIPBL

Santosh K. Gothwal[1#], Ahmed M. Refaat[1,2†], Mikiyo Nakata[1,2], Andre Stanlie[1ϕ], Tasuku Honjo[1,2*] and Nasim A. Begum[1,2]

[1]Department of Immunology and Genomic Medicine, Kyoto University Graduate School of Medicine, Kyoto 606-8501, Japan

[2]Center for Cancer Immunotherapy and Immunobiology, Kyoto University Graduate School of Medicine, Kyoto 606-8501, Japan

[†]Current address: Zoology Department, Faculty of Science, Minia University, El-Minia, 61519, Egypt

[#]Current address: Institute for Frontier Science Initiative, Kanazawa University, Ishikawa, 920-8640, Japan

[ϕ]Current address: Be Biopharma, Inc., Cambridge, MA 02139, USA

***Correspondence:** honjo@mfour.med.kyoto-u.ac.jp

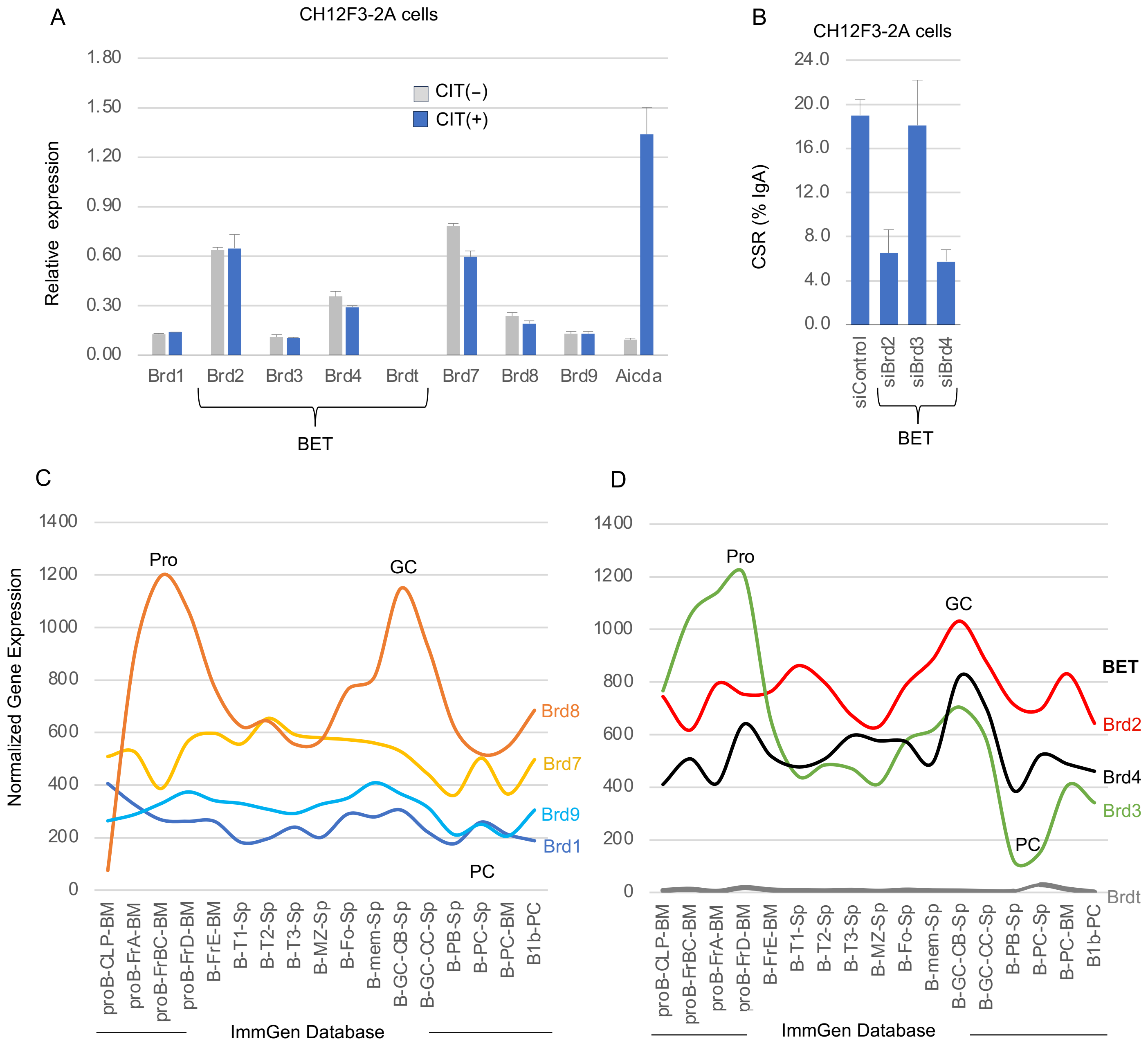

Supplementary Figure S1. Expression and screening of BET domain-containing genes in B cells. **(A)** The expression profile of selected BRD genes and AID (*Aicda*) in CH12F3-2A cells with and without CSR activation. Total RNA was isolated from cells stimulated with CIT for 24 hours. The BET group of BRD genes consists of *Brd2*, *Brd3*, *Brd4*, and *Brdt* (expressed exclusively in the testis). **(B)** Knockdown of *Brd2* and *Brd4*, but not *Brd3*, reduced IgM to IgA class switch in CH12F3-2A cells. **(C-D)** The expression of the BRD genes in various B cell populations (Immgen database: http://www.immgen.org). Germinal center (GC) B-cells show *Brd8*, *Brd2*, and *Brd4* expression, while pro-B cells show *Brd8* and *Brd3* expression. *Brd8* shows a unique profile with high pro and GC B-cell expression. *Brd3* appears to express exclusively in pro-B cells. No BRD genes seem to express in plasma cells (PC). **(A-B)** Error bars represent SD from two independent experiments.

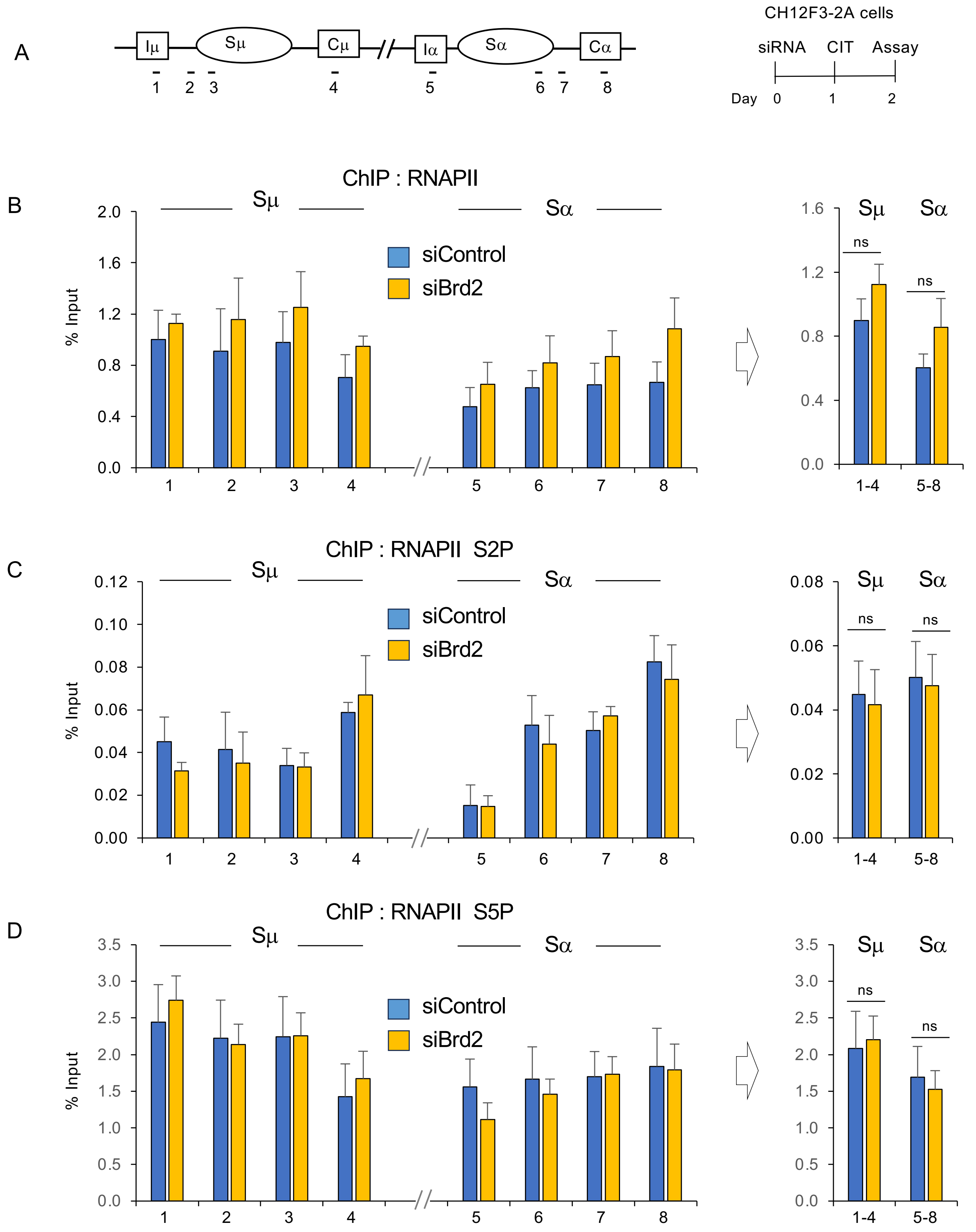

Supplementary Figure S2. BRD2 depletion does not affect RNA Pol II (RNAPII) distribution in the recombining S regions during CSR. **(A)** Left: Schematic representation of the *IgH* locus with the positions of the ChIP-qPCR amplicons numbered 1-8 (small bars). Right: The timeline of siRNA transfection, CSR induction, and cell harvest is the same as Fig.1 A-C. **(B-D)** The ChIP results of RNAPII **(B)**, RNAPII S2P **(C)**, and RNAPII S5P **(D)**; the plot on the right of each panel displays the cumulative profile of respective RNAPII on Sμ and Sα. All graphs show mean values ± SD. The statistical significance was calculated using a two-tailed Student's t-test of unequal variance (ns, non-significant).

```
   1 ATGGTGAGCAAGGGCGAGGAGCTGTTCACCGGGGTGGTGCCCATCCTGGTCGAGCTGGACGGCGACGTAAACGGCCACAAGTTCAGCGTG EGFP
     M  V  S  K  G  E  E  L  F  T  G  V  V  P  I  L  V  E  L  D  G  D  V  N  G  H  K  F  S  V
  91 TCCGGCGAGGGCGAGGGCGATGCCACCTACGGCAAGCTGACCCTGAAGTTCATCTGCACCACCGGCAAGCTGCCCGTGCCCTGGCCCACC
     S  G  E  G  E  G  D  A  T  Y  G  K  L  T  L  K  F  I  C  T  T  G  K  L  P  V  P  W  P  T
 181 CTCGTGACCACCCTGACCTACGGCGTGCAGTGCTTCAGCCGCTACCCCGACCACATGAAGCAGCACGACTTCTTCAAGTCCGCCATGCCC
     L  V  T  T  L  T  Y  G  V  Q  C  F  S  R  Y  P  D  H  M  K  Q  H  D  F  F  K  S  A  M  P
 271 GAAGGCTACGTCCAGGAGCGCACCATCTTCTTCAAGGACGACGGCAACTACAAGACCCGCGCCGAGGTGAAGTTCGAGGGCGACACCCTG
     E  G  Y  V  Q  E  R  T  I  F  F  K  D  D  G  N  Y  K  T  R  A  E  V  K  F  E  G  D  T  L
 361 GTGAACCGCATCGAGCTGAAGGGCATCGACTTCAAGGAGGACGGCAACATCCTGGGGCACAAGCTGGAGTACAACTACAACAGCCACAAC
     V  N  R  I  E  L  K  G  I  D  F  K  E  D  G  N  I  L  G  H  K  L  E  Y  N  Y  N  S  H  N
 451 GTCTATATCATGGCCGACAAGCAGAAGAACGGCATCAAGGTGAACTTCAAGATCCGCCACAACATCGAGGACGGCAGCGTGCAGCTCGCC
     V  Y  I  M  A  D  K  Q  K  N  G  I  K  V  N  F  K  I  R  H  N  I  E  D  G  S  V  Q  L  A
 541 GACCACTACCAGCAGAACACCCCCATCGGCGACGGCCCCGTGCTGCTGCCCGACAACCACTACCTGAGCACCCAGTCCGCCCTGAGCAAA
     D  H  Y  Q  Q  N  T  P  I  G  D  G  P  V  L  L  P  D  N  H  Y  L  S  T  Q  S  A  L  S  K
 631 GACCCCAACGAGAAGCGCGATCACATGGTCCTGCTGGAGTTCGTGACCGCCGCCGGGATCACTCTCGGCATGGACGAGCTGTACAAGTCC
     D  P  N  E  K  R  D  H  M  V  L  L  E  F  V  T  A  A  G  I  T  L  G  M  D  E  L  Y  K  S
 721 GGCCGGACTCAGATCTCGAGCTCAAGCTTCGAATTCCTGCAAAACGTGACTCCCCACAAGCTCCCTGGGGAAGGGAATGCAGGGTTACTG
     G  R  T  Q  I  S  S  S  S  F  E  F  L  Q  N  V  T  P  H  K  L  P  G  E  G  N  A  G  L  L
 811 GGGCTGGGCCCAGAGGCAGCAGCACCAGGGAAAAGGATTCGAAAGCCTTCTCTGCTGTATGAGGGATTTGAGAGCCCCACAATGGCTTCT
     G  L  G  P  E  A  A  A  P  G  K  R  I  R  K  P  S  L  L  Y  E  G  F  E  S  P  T  M  A  S
 901 GTACCAGCTTTACAACTGGCCCCTGCCAATCCACCACCCCCTGAGGTGTCCAATCCCAAAAAGCCAGGACGGGTAACAAACCAACTGCAG   BD1
     V  P  A  L  Q  L  A  P  A  N  P  P  P  P  E  V  S  N  P  K  K  P  G  R  V  T  N  Q  L  Q
 991 TACCTGCACAAGGTAGTGATGAAGGCTCTGTGGAAGCATCAGTTTGCATGGCCATTCCGGCAGCCTGTGGACGCTGTGAAGCTGGGTTTG
     Y  L  H  K  V  V  M  K  A  L  W  K  H  Q  F  A  W  P  F  R  Q  P  V  D  A  V  K  L  G  L
1081 CCGGATTATCACAAAATTATAAAACAGCCTATGGACATGGGTACTATCAAGAGGAGACTTGAAAACAATTACTACTGGGCTGCCTCAGAA
     P  D  Y  H  K  I  I  K  Q  P  M  D  M  G  T  I  K  R  R  L  E  N  N  Y  Y  W  A  A  S  E
1171 TGTATGCAGGACTTTAATACTATGTTTACCAACTGTTACATTTATAACAAGCCCACCGATGATATTGTCCTAATGGCACAGACACTGGAA
     C  M  Q  D  F  N  T  M  F  T  N  C  Y  I  Y  N  K  P  T  D  D  I  V  L  M  A  Q  T  L  E
1261 AAGATCTTCCTACAGAAAGTGGCATCCATGCCACAAGAGGAGCAAGAGCTTGTGGTGACCATCCCTAAAAACAGCCATAAGAAGGGGGCC
     K  I  F  L  Q  K  V  A  S  M  P  Q  E  E  Q  E  L  V  V  T  I  P  K  N  S  H  K  K  G  A
1351 AAGTTAGCAGCACTCCAGGGCAGTATTACCAGTGCCCATCAGGTGCCTGCTGTCTCTTCTGTGTCGCATACAGCCCTGTATACACCACCA
     K  L  A  A  L  Q  G  S  I  T  S  A  H  Q  V  P  A  V  S  S  V  S  H  T  A  L  Y  T  P  P

1441 CCTGAAATACCTACCACTGTCCTCAACATTCCCCATCCGAGTGTAATTAGCAGCCCATTACTTAAGTCCCTGCATTCTGCTGGACCCCCA  Brd2[R]
     P  E  I  P  T  T  V  L  N  I  P  H  P  S  V  I  S  S  P  L  L  K  S  L  H  S  A  G  P  P
1531 CTCCTTGCTGTATCAGCAGCGCCTCCAGCTCAGCCCCTTGCCAAGAAAAAGGCGTTAAACGGAAAGCGGATACTACCACCCCTACACCC
     L  L  A  V  S  A  A  P  P  A  Q  P  L  A  K  K  K  G  V  K  R  K  A  D  T  T  T  P  T  P
1621 ACAGCCATCCTGGCTCCTGGTTCCCCAGCTAGTCCTCCTGGGAGTCTTGAGCCAAAGGCAGCAAGGCTCCCTCCTATGCGCAGAGAGAGT
     T  A  I  L  A  P  G  S  P  A  S  P  P  G  S  L  E  P  K  A  A  R  L  P  P  M  R  R  E  S
1711 GGCCGCCCAATCAAACCCCCACGAAAAGACTTGCCTGACTCGCAACAGCAACACCAGAGCTCTAAGAAAGGGAAGCTGTCAGAGCAGTTA  BD2
     G  R  P  I  K  P  P  R  K  D  L  P  D  S  Q  Q  Q  H  Q  S  S  K  K  G  K  L  S  E  Q  L
1801 AAGCACTGCAACGGCATCCTGAAGGAACTGCTCTCAAAGAAGCACGCTGCCTACGCCTGGCCCTTCTATAAGCCAGTGGACGCTTCTGCT
     K  H  C  N  G  I  L  K  E  L  L  S  K  K  H  A  A  Y  A  W  P  F  Y  K  P  V  D  A  S  A
1891 CTTGGCCTTCATGATTACCATGACATCATTAAACACCCCATGGACCTCAGCACTGTCAAGCGGAAGATGGAGAACCGTGACTACCGGGAT
     L  G  L  H  D  Y  H  D  I  I  K  H  P  M  D  L  S  T  V  K  R  K  M  E  N  R  D  Y  R  D
1981 GCACAGGAGTTTGCTGCTGATGTACGGCTTATGTTCTCCAACTGCTATAAGTACAATCCTCCAGACCACGATGTTGTGGCTATGGCACGA
     A  Q  E  F  A  A  D  V  R  L  M  F  S  N  C  Y  K  Y  N  P  P  D  H  D  V  V  A  M  A  R
2071 AAGTTGCAGGATGTGTTTGAGTTTCGCTATGCCAAGATGCCAGATGAGCCACTGGAACCAGGACCTCTACCAGTCTCTACTGCCTTGCCT
     K  L  Q  D  V  F  E  F  R  Y  A  K  M  P  D  E  P  L  E  P  G  P  L  P  V  S  T  A  L  P
2161 CCTGGGTTGACCAAATCCTCTTCAGAGTCCTCCAGTGAGGAAAGTAGCAGTGAGAGTTCCTCTGAGGAAGAGGAGGAGGAGGAAGAAGAT
     P  G  L  T  K  S  S  S  E  S  S  S  E  E  S  S  S  E  S  S  S  E  E  E  E  E  E  E  E  D
2251 GAGGACGAGGAGGAGAGTGAAAGCTCAGACTCTGAGGAGGAAAGGGCTCATCGCCTAGCAGAGCTGCAGGAGCAGCTTCGGGCAGTTCAT
     E  D  E  E  E  S  E  S  S  D  S  E  E  E  R  A  H  R  L  A  E  L  Q  E  Q  L  R  A  V  H
2341 GAACAACTGGCTGCCCTGTCCCAGGGCCCAATATCTAAGCCCAAGCGGAAGAGAGAGAAAAAGGAAAAAAAGAAGAAACGGAAGGCAGAG  NLS
     E  Q  L  A  A  L  S  Q  G  P  I  S  K  P  K  R  K  R  E  K  K  E  K  K  K  K  R  K  A  E
2431 AAACATCGTGGCCGAATTGGGATCGATGAAGATGATAAGGGGCCTAGGGCACCTCGCCCACCTCAGCCCAAGAAATCTAAGAAAGCAGGT
     K  H  R  G  R  I  G  I  D  E  D  D  K  G  P  R  A  P  R  P  P  Q  P  K  K  S  K  K  A  G
2521 GGTGGGGGTAGCAATGCTACTACACTCAGCCATCCTGGCTTTGGGACTTCCGGAGGAAGTAGCAACAAGCTACCTAAAAAGTCTCAAAAG
     G  G  G  S  N  A  T  T  L  S  H  P  G  F  G  T  S  G  G  S  S  N  K  L  P  K  K  S  Q  K
2611 ACAGCTCCACCTGTCCTTCCCACTGGCTATGATTCTGAGGAGGAGGAAGAAAGCAGGCCCATGAGTTATGATGAGAAGAGACAGTTAAGC  ET
     T  A  P  P  V  L  P  T  G  Y  D  S  E  E  E  E  E  S  R  P  M  S  Y  D  E  K  R  Q  L  S
2701 CTGGATATCAATAAGTTACCTGGGGAAAAGCTGGGTCGAGTAGTACATATCATCCAAGCCAGGGAACCCTCTCTACGTGATTCAAATCCA
     L  D  I  N  K  L  P  G  E  K  L  G  R  V  V  H  I  I  Q  A  R  E  P  S  L  R  D  S  N  P
2791 GAAGAAATTGAGATTGATTTTGAAACACTCAAGCCGTCCACACTTAGAGAGCTTGAGCGATATGTTTTATCCTGCCTTCGAAAGAAACCC
     E  E  I  E  I  D  F  E  T  L  K  P  S  T  L  R  E  L  E  R  Y  V  L  S  C  L  R  K  K  P
2881 CGGAAGCCCTACACTATTAGGAAACCTGTGGGAAAAACAAAGGAGGAACTGGCTTTGGAGAAGAAGCGGGAGCTAGAGAAGCGGTTGCAG
     R  K  P  Y  T  I  R  K  P  V  G  K  T  K  E  E  L  A  L  E  K  K  R  E  L  E  K  R  L  Q
2971 GATGTCAGTGGACAGCTCAACTCCACCAAAAAGCCTCCCAAGAAAGCGAGTGAGAAGACAGAGTCATCTGCACAGCAAGTGGCAGTGTCC
     D  V  S  G  Q  L  N  S  T  K  K  P  P  K  K  A  S  E  K  T  E  S  S  A  Q  Q  V  A  V  S
3061 CGTCTCAGTGCTTCTAGTTCCAGCTCAGATTCCAGCTCCTCGTCGTCATCTTCCTCTTCTTCAGACACCAGCGATTCAGACTCGGGCACG
     R  L  S  A  S  S  S  S  S  D  S  S  S  S  S  S  S  S  S  S  S  D  T  S  D  S  D  S  G  T
3151 CGTACGCGGCCGCTCGAGCAGAAACTCATCTCAGAAGAGGATCTGGCAGCAAATGATATCCTGGATTACAAGGATGACGACGATAAGGTT
     R  T  R  P  L  E  Q  K  L  I  S  E  E  D  L  A  A  N  D  I  L  D  Y  K  D  D  D  D  K  V
3241 TAA                 [MYC]                                          [FLAG]
     *
```

Supplementary Figure S3. The sequence of *Brd2* expression construct used in CSR complementation assay. The wild-type mouse BRD2 expression construct used in the CSR complementation assay (Fig.1G) illustrates the arrangement of nucleotides encoding various functional domains and elements. EGFP was fused at the N-terminus, and Myc-FLAG (shaded in pink) was tagged at the C-terminus. The sequences colored in red have been mutated to prevent the degradation of the transcribed mRNA by siBrd2 and denoted as *Brd2*^{R}. The BD1 and BD2 domains (shaded in light blue) and the ET domain (shaded in light purple) are highlighted within the sequence. The nuclear localization signal (NLS) motif is positioned between the BD2 and ET domains. All the constructs used have an intact NLS (Fig. 1G and Fig. 5B)

A

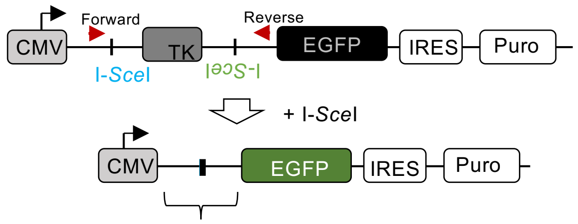

I-*Sce*I-induced DSB end-joining event analysis

<table>
<tr><th>siRNA</th><th>DSB1 (I-Sce I)
ATCTAGGGATAA|CAGGGTAAT</th><th>MH; Insertion</th><th>DSB2 (I-Sce I)
ATTACCCTGTTAT|CCCTAGGATCCTTCC</th><th>Clone no.</th></tr>
<tr><td>siControl (+I-Sce I)</td><td>ATCTAGGGATA</td><td>A</td><td>T|CCCTAGGATCCTTCC</td><td>14</td></tr>
<tr><td></td><td>ATCTAGGGA</td><td>T</td><td>TAT|CCCTAGGATCCTTCC</td><td>16</td></tr>
<tr><td></td><td>ATCTAGGG</td><td>A</td><td>GGATCCTTCC</td><td>1</td></tr>
<tr><td></td><td>ATCTAGGGA</td><td>T</td><td>TCC</td><td>1</td></tr>
<tr><td></td><td>ATCTAGGGA</td><td>T</td><td>|CCCTAGGATCCTTCC</td><td>1</td></tr>
<tr><td></td><td>ATCTAGGGA</td><td>G</td><td>TTAT|CCCTAGGATCCTTCC</td><td>1</td></tr>
<tr><td></td><td>ATCTAGGGA</td><td>TA</td><td>T|CCCTAGGATCCTTCC</td><td>5</td></tr>
<tr><td></td><td>ATC</td><td>TA</td><td>T|CCCTAGGATCCTTCC</td><td>3</td></tr>
<tr><td></td><td>ATCTAGGG</td><td>AT</td><td>|CCCTAGGATCCTTCC</td><td>3</td></tr>
<tr><td></td><td>Δ104</td><td>CC</td><td>CTAGGATCCTTCC</td><td>1</td></tr>
<tr><td></td><td>ATCTAG</td><td>GGA</td><td>TCCTTCC</td><td>1</td></tr>
<tr><td></td><td>Δ113</td><td>TAT</td><td>|CCCTAGGATCCTTCC</td><td>1</td></tr>
<tr><td></td><td>ATCTAGGG</td><td></td><td>Δ124</td><td>1</td></tr>
<tr><td></td><td>ATCTAGGGA</td><td>In1</td><td>Δ114</td><td>1</td></tr>
<tr><td></td><td>ATCTAGGGATAA</td><td>In1</td><td>T|CCCTAGGATCCTTCC</td><td>1</td></tr>
<tr><td></td><td>ATCTAGGGAT</td><td>In3</td><td>Δ141</td><td>1</td></tr>
<tr><td></td><td>ATCTAGGGATAA|</td><td>In4</td><td>TAT|CCCTAGGATCCTTCC</td><td>1</td></tr>
<tr><td></td><td>ATCTAGGGATAA|</td><td>In17</td><td>TAT|CCCTAGGATCCTTCC</td><td>1</td></tr>
<tr><td></td><td>ATCTAGGGATAA|</td><td>In19</td><td>Δ166</td><td>1</td></tr>
<tr><td></td><td></td><td></td><td></td><td>n= 55</td></tr>
<tr><td>siBrd2 (+I-Sce I)</td><td>ATCTAGGGATA</td><td>A</td><td>T|CCCTAGGATCCTTCC</td><td>6</td></tr>
<tr><td></td><td>ATCTAGGGA</td><td>T</td><td>TAT|CCCTAGGATCCTTCC</td><td>5</td></tr>
<tr><td></td><td>ATC</td><td>T</td><td>TAT|CCCTAGGATCCTTCC</td><td>1</td></tr>
<tr><td></td><td>ATCTAGGGATAA|</td><td>C</td><td>Δ121</td><td>1</td></tr>
<tr><td></td><td>AT</td><td>C</td><td>ATTACCCTGTTAT|CCCTAGGATCCTTCC</td><td>1</td></tr>
<tr><td></td><td>ATCTAGGGA</td><td>TA</td><td>T|CCCTAGGATCCTTCC</td><td>3</td></tr>
<tr><td></td><td>ATCTAGGG</td><td>AT</td><td>|CCCTAGGATCCTTCC</td><td>1</td></tr>
<tr><td></td><td></td><td>ATC</td><td>CCTAGGATCCTTCC</td><td>1</td></tr>
<tr><td></td><td>Δ160</td><td>CTG</td><td>TTAT|CCCTAGGATCCTTCC</td><td>1</td></tr>
<tr><td></td><td>ATCTAGG</td><td>GAT</td><td>Δ97</td><td>1</td></tr>
<tr><td></td><td>ATCTAGGGATAA|CAGGGTAAT</td><td>TTAT</td><td>TTAT|CCCTAGGATCCTTCC</td><td>1</td></tr>
<tr><td></td><td>ATCTAGGGATAA|CAGGGTAAT</td><td>GGCA</td><td>Δ116</td><td>1</td></tr>
<tr><td></td><td>Δ15</td><td>ATTC</td><td>CTAGGATCCTTCC</td><td>1</td></tr>
<tr><td></td><td>ATCTAGGGATAA|</td><td>CAGGG</td><td>Δ174</td><td>1</td></tr>
<tr><td></td><td>Δ134</td><td>TTATC</td><td>CCTAGGATCCTTCC</td><td>2</td></tr>
<tr><td></td><td>ATCTAGGGATA</td><td>AGAGGG</td><td>Δ157</td><td>1</td></tr>
<tr><td></td><td>ATCTAGGGA</td><td>AAGCTT;In5</td><td>ATTACCCTGTTAT|CCCTAGGATCCTTCC</td><td>1</td></tr>
<tr><td></td><td>ATCTAGGGATAA|CAGGGTAAT</td><td>GCCACCATG</td><td>Δ75</td><td>2</td></tr>
<tr><td></td><td>ATCTAGGGATAA|</td><td>CAGGGTAATGGC;In154</td><td>TTAT|CCCTAGGATCCTTCC</td><td>1</td></tr>
<tr><td></td><td>ATCTAGGGATAA|CAGGG</td><td>In69</td><td>CTGTTAT|CCCTAGGATCCTTCC</td><td>1</td></tr>
<tr><td></td><td>ATCTAGGGATAA|</td><td>In86</td><td>TTAT|CCCTAGGATCCTTCC</td><td>1</td></tr>
<tr><td></td><td>ATCTAGGG</td><td>In127</td><td>AT|CCCTAGGATCCTTCC</td><td>1</td></tr>
<tr><td></td><td>ATCTAGGGA</td><td>In214</td><td>TTAT|CCCTAGGATCCTTCC</td><td>1</td></tr>
<tr><td></td><td>ATCTAGGGAT</td><td>In251</td><td>TTAT|CCCTAGGATCCTTCC</td><td>1</td></tr>
<tr><td></td><td>ATCTAGGGATAA|</td><td>In106</td><td>|CCCTAGGATCCTTCC</td><td>1</td></tr>
<tr><td></td><td>ATCTAGGGATAA|C</td><td>In120</td><td>TAT|CCCTAGGATCCTTCC</td><td>1</td></tr>
<tr><td></td><td>ATCTAGGGA</td><td>In125</td><td>TTAT|CCCTAGGATCCTTCC</td><td>1</td></tr>
<tr><td></td><td>ATCTAGGGATAA|</td><td>In151</td><td>TTAT|CCCTAGGATCCTTCC</td><td>1</td></tr>
<tr><td></td><td>ATCTAGGGA</td><td>In249</td><td>T|CCCTAGGATCCTTCC</td><td>1</td></tr>
<tr><td></td><td>ATCTAGGGATAA|</td><td>In303</td><td>TTAT|CCCTAGGATCCTTCC</td><td>1</td></tr>
<tr><td></td><td>ATCTAGGGAT</td><td>In304</td><td>TTAT|CCCTAGGATCCTTCC</td><td>1</td></tr>
<tr><td></td><td>ATCTAGGGATAA|C</td><td>In369</td><td>T|CCCTAGGATCCTTCC</td><td>1</td></tr>
<tr><td></td><td>ATCTAGGGATAA|</td><td>In393</td><td>TTAT|CCCTAGGATCCTTCC</td><td>1</td></tr>
<tr><td></td><td>ATCTAGGGATAA|</td><td>In422</td><td>TTAT|CCCTAGGATCCTTCC</td><td>1</td></tr>
<tr><td></td><td></td><td></td><td></td><td>n= 47</td></tr>
</table>

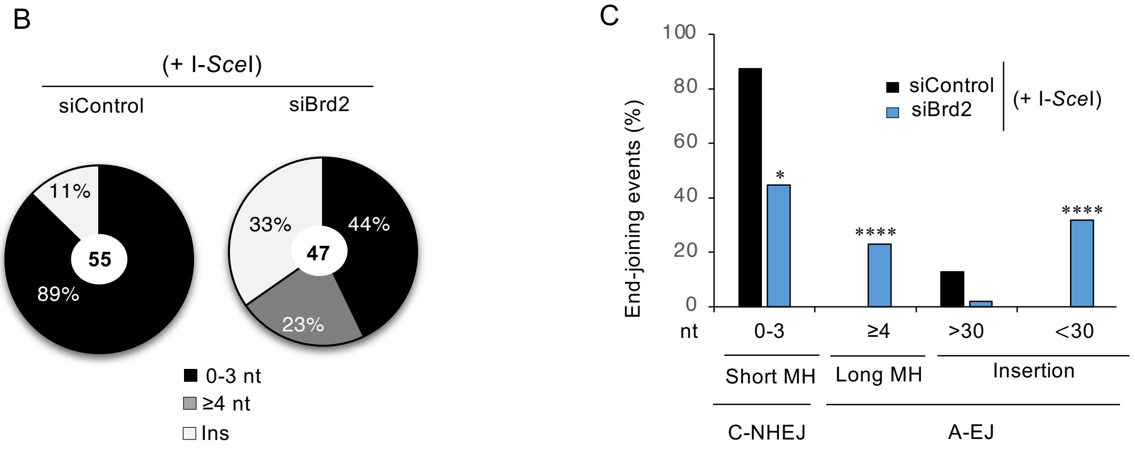

Supplementary Figure S4. Impaired NHEJ-mediated repair of the I-*Sce*I-induced DSBs upon the absence of BRD2. **(A)** Sequence analysis of I-*Sce*I-induced Double-Strand Break (DSB) end-joining events in BRD2 depleted NHEJ reporter cell line. Genomic DNA was isolated from cells treated with siControl and siBrd2, followed by PCR amplification of the repaired junctions using primers flanking the two DSB sites. The two recognition sequences for I-*Sce*I are 18 nucleotides long, colored in green and cyan, and bolded. The I-*Sce*I cutting site is indicated by a bar, and microhomologies (MH) are in red. The nucleotide insertions (In) are black, and long deletions (Δ) are purple. **(B)** Pie charts comparing the junction patterns characterized by zero to short (0–3 nt) MH and longer (≥4 nt) MH along with nucleotide insertions. The number of junctions analyzed is indicated in the center of each chart. **(C)** The percentage of repaired junctions with the indicated length of MH or insertions was calculated and grouped under C-NHEJ or A-EJ**.** Statistical significance was calculated with a two-tailed Fisher's exact test (*$p \leq 0.05$, ****$p \leq 0.0001$). For this study, pooled genomic DNA from three experiments was used.

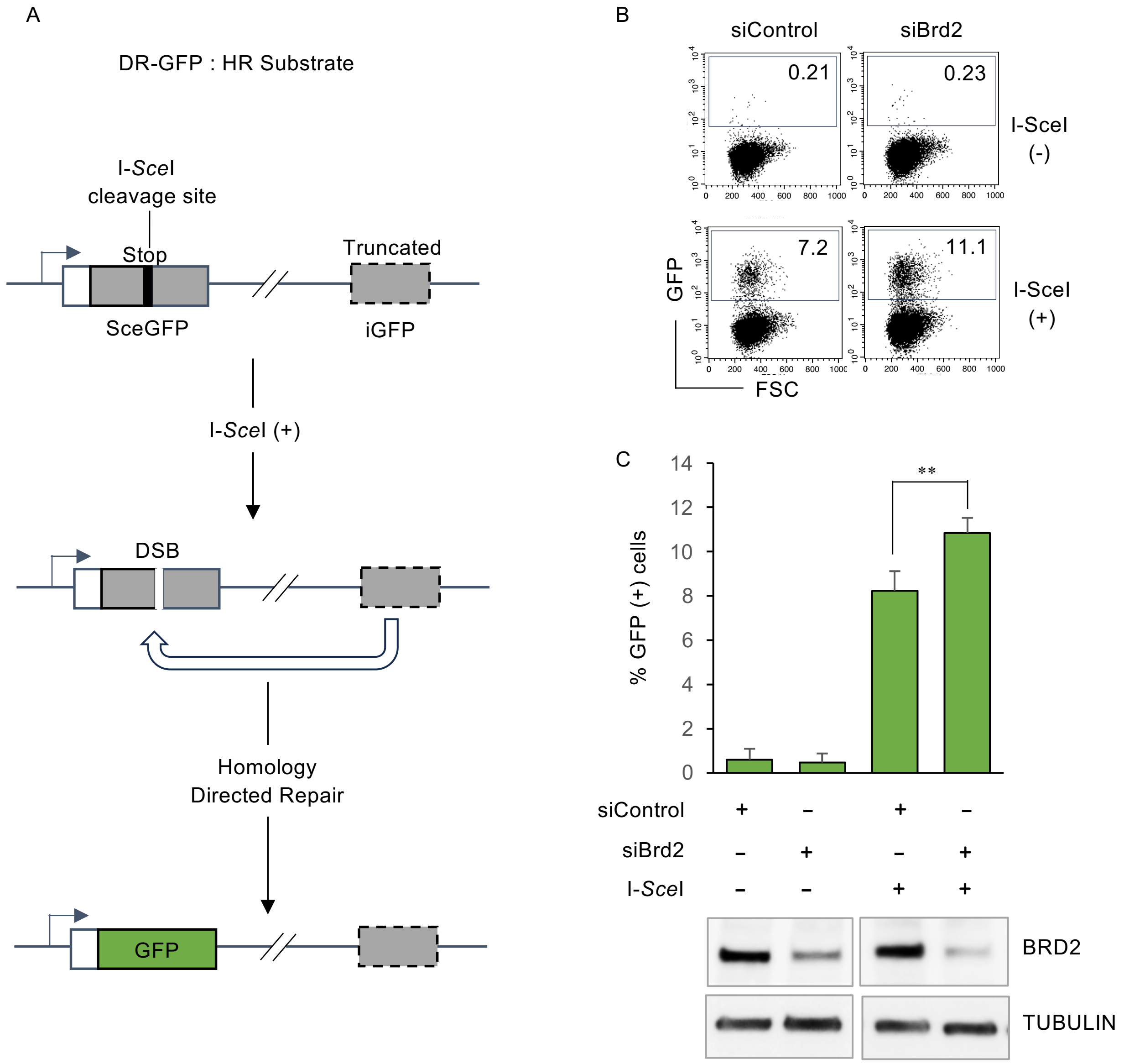

Supplementary Figure S5. Depletion of BRD2 does not impede DNA repair by homologous recombination (HR). **(A)** Schematic representation of the HR assay utilizing DR-GFP reporter. **(B)** CH12F3-2A cells, with stably integrated DR-GFP were transfected with indicated siRNAs along with I-*Sce*I (+) or empty vector (-). After 24 hours of transfection, GFP-positive cells were quantified by flow cytometry, and a representative result from four experiments is shown. **(C)** Top: Summarized result of GFP-positive cells from four independent HR assays. The error bars represent the standard deviation from the mean (**$p \leq 0.01$). Bottom: Representative results of immunoblots of whole cell extracts from the transfected cells with control siRNA or siRNA targeting *Brd2*.

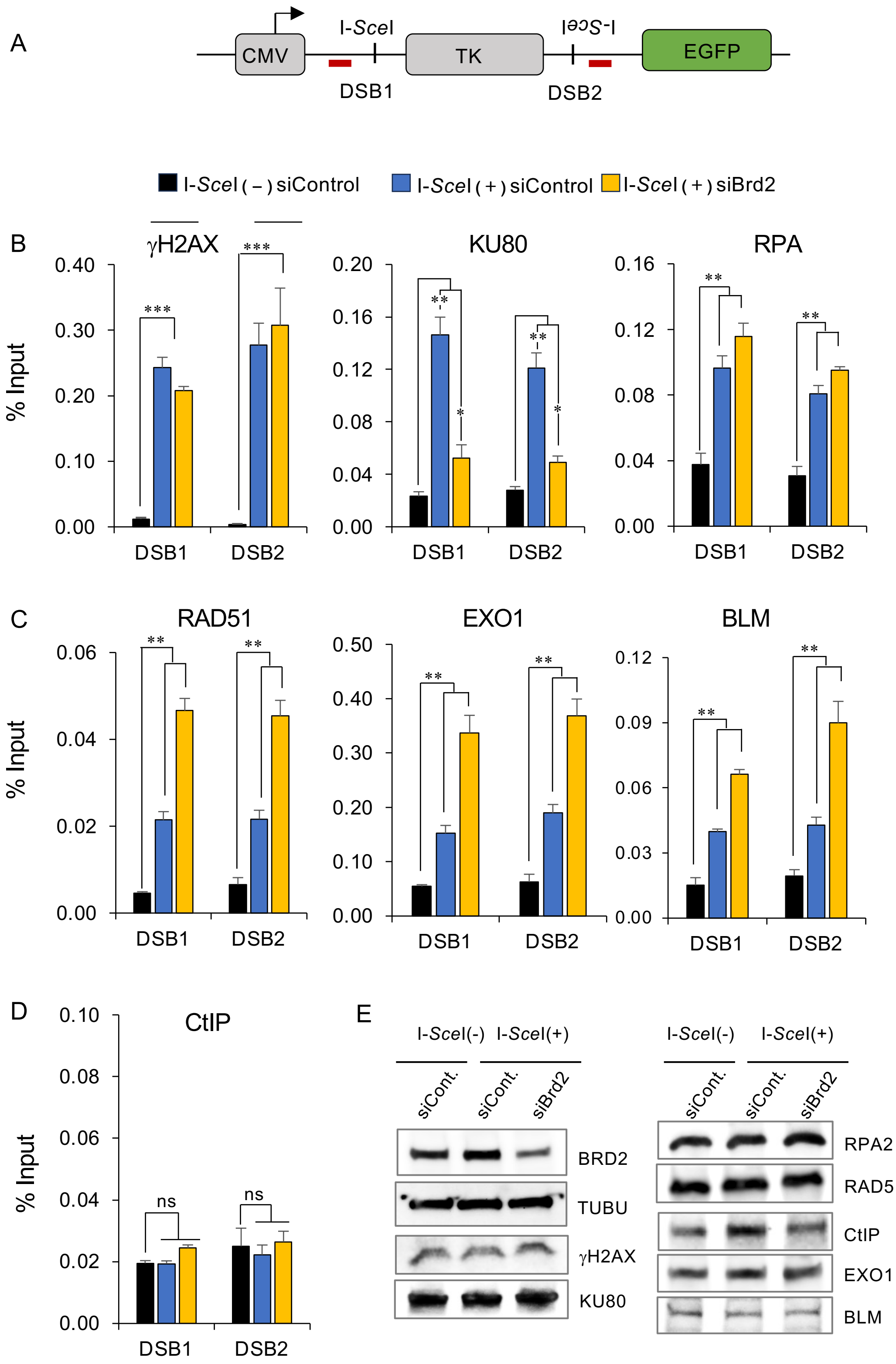

Supplementary Figure S6. BRD2 depletion affects KU80, RAD51, and EXO1 occupancy at I-*Sce*I-induced DSB sites. **(A)** The schematic of the NHEJ reporter construct displays two I-*Sce*I-induced DSB sites (DSB-1 and DSB-2). Repair of these DSBs results in the loss of the intervening Thymidine Kinase (TK) gene and subsequent EGFP expression. The red bars adjacent to each DSB site indicate the positions of PCR amplicons analyzed in the ChIP assay (B-D). Analysis of DNA damage response and resection-associated proteins in the NHEJ reporter cell line transfected with siRNA Control or siBrd2, both in the presence and absence of I-*Sce*I expression (B-E). **(B-D)** ChIP analyses show the occupancy of indicated proteins around DSB1 and DSB2 sites. Statistical significance was determined using a two-tailed Student's t-test of unequal variance (n=3 ±SD; *p ≤ 0.05; ** p ≤0.01; ***p ≤0.001; ns, non-significant). **(E)** A representative Western blot analysis demonstrates BRD2 depletion and expression of the proteins analyzed by ChIP (B-E).

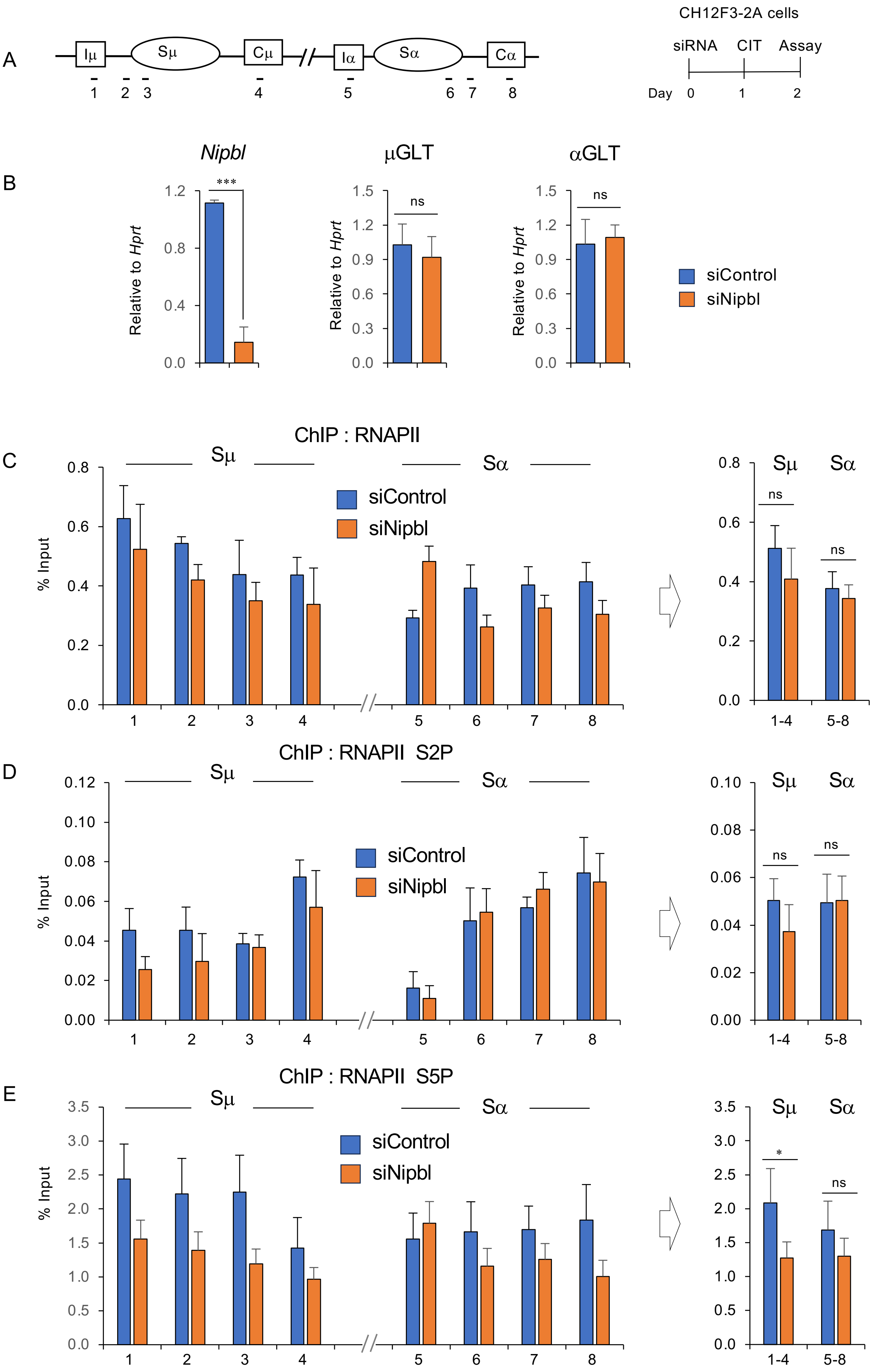
A
Iμ
Sμ
Cμ
Iα
Sα
Cα
1 2 3 4 5 6 7 8
CH12F3-2A cells
siRNA CIT Assay
Day 0 1 2
B
Nipbl
μGLT
αGLT
Relative to Hprt
***
ns
ns
siControl
siNipbl
C
ChIP : RNAPII
% Input
Sμ
Sα
ns
ns
1-4
5-8
D
ChIP : RNAPII S2P
% Input
siControl
siNipbl
ns
ns
E
ChIP : RNAPII S5P
% Input
siControl
siNipbl
*
ns

Supplementary Figure S7. Knockdown of *Nipbl* did not perturb RNA Pol II (RNAPII) distribution in the S regions. **(A)** Left: Schematic representation of the *IgH* locus highlighting Sμ and Sα. The small bars below the diagram indicate the positions of the ChIP-qPCR amplicons, numbered 1-8. Right: The assay timeline is the same as in Figure 5C-D. **(B)** RT-qPCR result shows the *Nipbl* knockdown efficiency and the status of μGLT and αGLT under identical conditions. **(C-E)** ChIP analysis of RNAPII (C), RNAPII S2P (D), and RNAPII S5P (E) occupancy. The plot on the right of each panel displays the cumulative profile of respective RNAPII surrounding Sμ and Sα. (B-E) The data represents mean ± SD (n= 3-4). Statistical significance was determined by a two-tailed Student's t-test of unequal variance. *$p \leq 0.05$; ***$p \leq 0.001$; ns, non-significant.

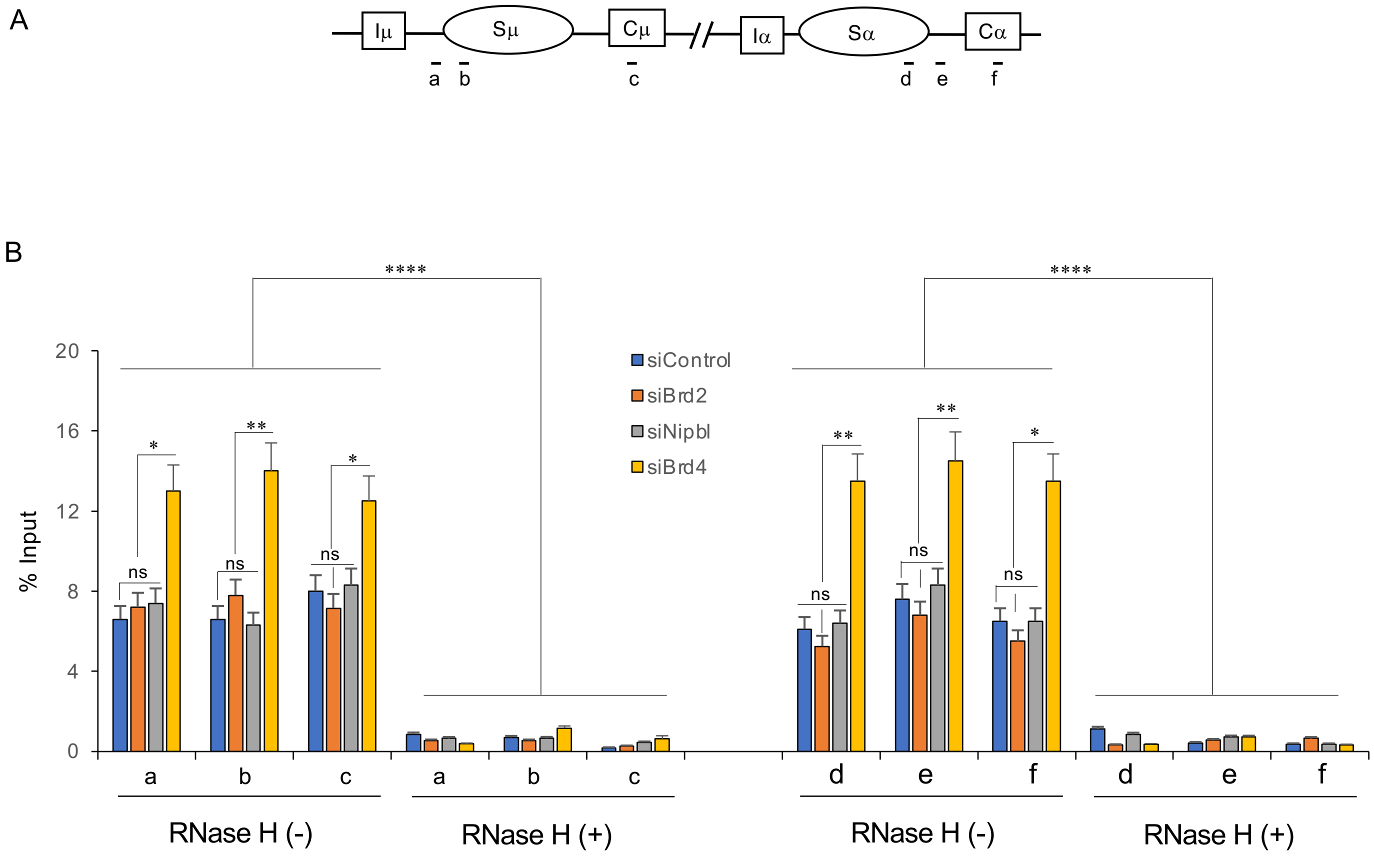

Supplementary Figure S8. Knockdown of *Brd4* but not *Brd2* or *Nipbl* elevates S region R-loop. The R-loop or the DNA: RNA hybrid immunoprecipitation (DRIP) was performed using the S9.6 antibody in CH12F3-2A cells. **(A)** Schematic representation of IgH locus centering Sμ and Sα. The small bars below the diagram, denoted by a-f, show the positions of qPCR amplicons. **(B)** DRIP assay was conducted using CH12F3-2A cells transfected with indicated siRNAs and activated for CSR, as in Fig.6A-H. All genomic DNA samples were prepared with RNase A treatment, and each sample was divided into two halves for RNaseH (+) and without RNaseH (-) treatments. Both groups were then subjected to IP using the S9.6 antibody, and the background signals from the control IgG antibody were subtracted. To calculate the DRIP percentage, the values were normalized to the total input DNA Data show means ± SD (n=3). Statistical analysis was performed by Student's t-test (* $p \leq 0.05$; ** $p \leq 0.01$; ns, non-significant) and two-way ANOVA (**** $p \leq 0.0001$).

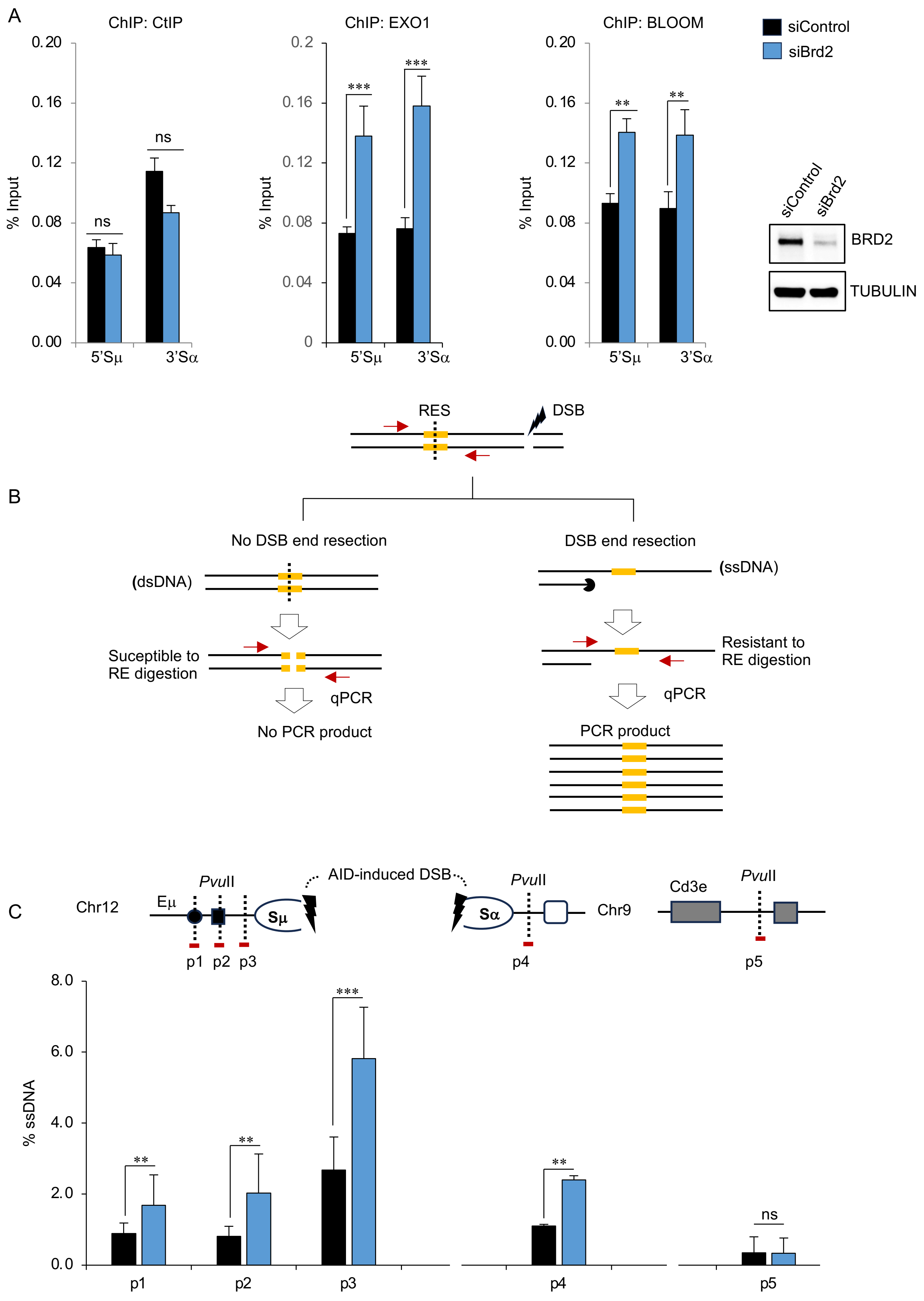
A
ChIP: CtIP
ChIP: EXO1
ChIP: BLOOM
siControl
siBrd2
% Input
0.20
0.16
0.12
0.08
0.04
0.00
0.2
0
ns
***
**
5'Sμ
3'Sα
siControl
siBrd2
BRD2
TUBULIN
RES
DSB
B
No DSB end resection
DSB end resection
(dsDNA)
(ssDNA)
Suceptible to
RE digestion
Resistant to
RE digestion
qPCR
No PCR product
PCR product
C
Chr12
Eμ
PvuII
Sμ
AID-induced DSB
Sα
Chr9
Cd3e
p1
p2
p3
p4
p5
% ssDNA
8.0
6.0
4.0
2.0
0.0

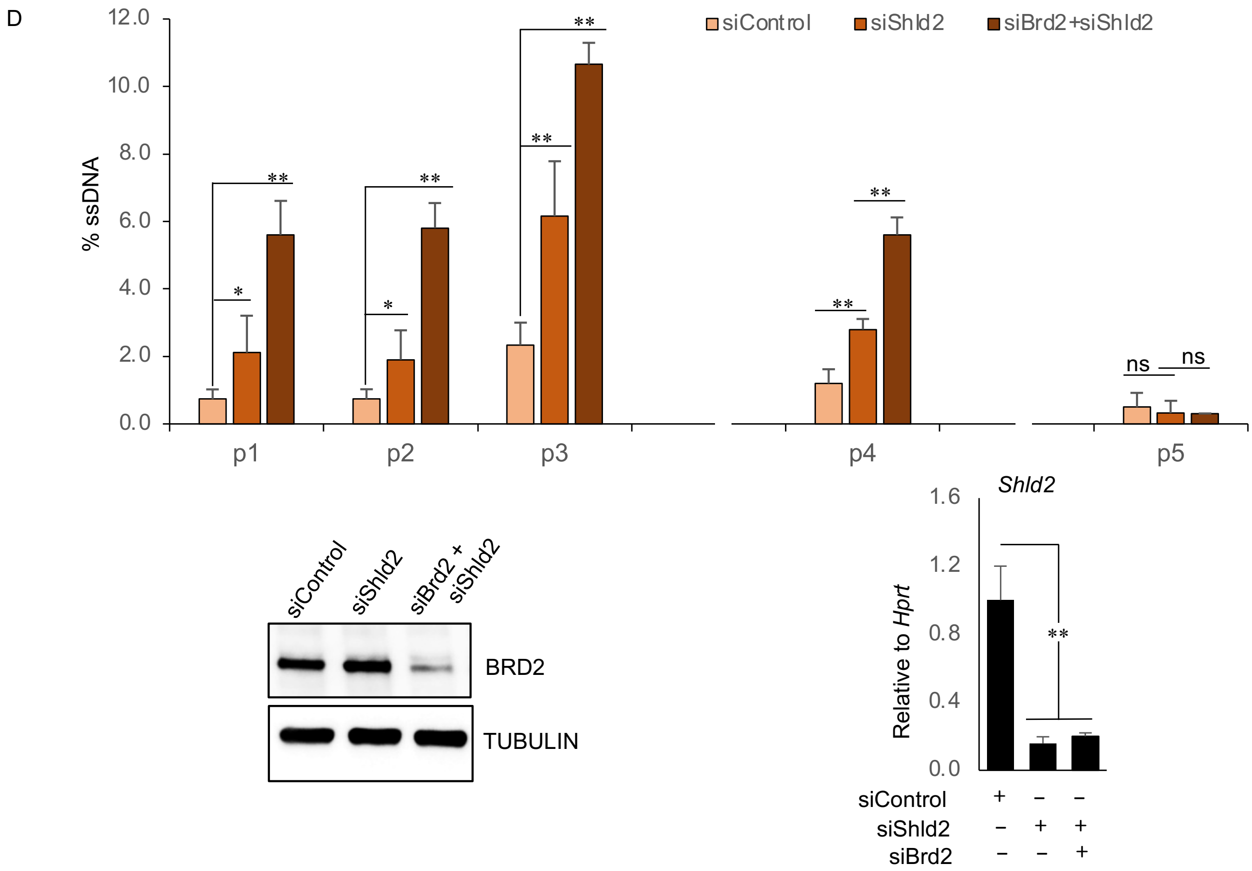

Supplementary Figure S9. BRD2 prevents DSB-end resection in the S region during CSR.

(A) Occupancy of the end resection components in CSR-activated CH12F3-2A cells transfected with siControl or siBrd2. A representative immunoblot using whole cell extracts of the transfected cells shows *Brd2* knockdown efficiency. ChIP assays were performed using indicated antibodies; bar graphs represent the ChIP qPCR result of CtIP, EXO1, and BLM occupancy in the recombining S regions.

(B) An illustration of quantitative DNA end resection assay. If the DSB is resected, the ssDNA will not be cut by the chosen restriction enzyme, and therefore, PCR products will be produced (right panel). On the other hand, if there is no DSB end processing, the RES will be intact and cut by the enzyme, resulting in no PCR product (left panel). RES, Restriction Endonuclease Site (yellow bar); dotted line, DNA cleavage by RE; ds, double strand; ss, single strand; arrows, primer positions; black crescent, indicates an exonucleolytic processing of the break-end.

(C) Top: Schematic representation of the recombining S regions in CH12F3-2A cells where AID-induced DSB occurs at Sμ and Sα (Chr 12). The Cd3e loci on Chr 9 is used as no DSB negative control. The positions of qPCR amplicons, each harboring a *Pvu*II site (dotted line), are denoted by p1-p5 with small bars above. Bottom: Assessment of DSB end resection in CH12F3-2A cells transfected with siControl or siBrd2. Genomic DNA isolated from siRNA-treated and CIT-stimulated cells was digested or mock-digested with *Pvu*II. DNA end resection adjacent to core Sm and Sα DSB (5'Sμ and 3'Sα) and the unrelated no DSB site (Cd3e) were measured by qPCR as described in the 'Materials and Methods' section.

(D) Assessment of S region DSB end resection in *Shld2* and *Brd2* and *Shld2* double knockdown in CH12F3-2A cells. In the bottom panel, the knockdown efficiency of *Brd2* and *Shld2* in the transfected cells was evaluated by immunoblotting whole cell lysate (left) and qRT-PCR of the total RNA (right), respectively.

(A-D) Error bars represent the standard deviation from the mean (n=3; ± SD). Statistical significance was measured by a two-tailed Student's t-test of unequal variance (*$p \leq 0.05$; **$p \leq 0.01$; ***$p \leq 0.001$; ns, non-significant).

Table S1. List of antibodies

Antibodies	Source	Catalog#	Usage
Anti-Mouse IgM-FITC	eBioscience	11-5890-85	FACS
Anti-Mouse IgA-PE	Southern Biotech	1040-09	FACS
Biotin Anti-Mouse IgG1	BD Pharmingen	553441	FACS
Streptavidine APC	eBioscience	17-4317	FACS
Rabbit IgG	Millipore	PP64B	ChIP
Anti-BRD2	Bethyl	A302-583A	ChIP, IP, IB
Anti-BRD4	Abcam	ab75898	ChIP, IP, IB
Anti-NIPBL	Bethyl	A301-779A	ChIP, IB
Anti-H4ac	Active Motif	39925	ChIP
Anti-γH2AX	Millipore	05-636	ChIP
Anti-Ku-80	Santa Cruz	sc-1485	ChIP
Anti-53BP1	Novus	NB100-304	ChIP
Anti-ZYMND8	Sigma	HPA020949	ChIP
Anti-L3MBTL1	Active Motif	39182	ChIP
Anti-MAD2L2/REV7	Abcam	ab180579	ChIP
Anti-RAD51	Abcam	ab176458	ChIP
Anti-RPA32/RPA2	Abcam	ab10359	ChIP
Anti-pRPA32 (Ser4, Ser8)	Bethyl	A300-245A	ChIP
Anti-CtIP	Active Motif	61942	ChIP
Anti-EXO1	Proteintech	16253-1-AP	ChIP
Anti-BLM	Abcam	ab2179	ChIP
Anti-RNA Pol II	Active Motif	39497	ChIP
Anti-RNA Pol II CTD phospho (Ser2)	Active Motif	61984	ChIP
Anti-RNA Pol II CTD phospho (Ser5)	Abcam	Ab5131	ChIP
Anti-DNA-RNA Hybrid [S9.6]	Kerafast	ENH001	DRIP
Anti-Mouse Ig HRP	Rockland	18-8817-33	IB
Anti-Rabbit-IgG HRP	Rockland	18-8816-33	IB
Anti-Tubulin	Calbiochem	CP06	IB
Anti-FLAG	Sigma	F-3165	IP, IB
Anti-AID	Cell Signaling Technology	4949s	IB
Anti-SMC1	Active Motif	61068	IB
Anti-SMC3	Active Motif	61132	IB
Anti-CTCF	Abcam	ab7030	IB
Anti-NIPBL	Proteintech	187921-AP	IP, IB

Table S2. List of primers and siRNAs

RT-qPCR	Primer Name	Sequence/ other information
μGLT	Forward<br>Reverse	5'-CTCTGGCCCTGCTTATTGTTG<br>5'-AATGGTGCTGGGCAGGAAGT
αGLT	Forward<br>Reverse	5'-CCTGGCTGTTCCCCTATGAA<br>5'-GAGCTGGTGGGAGTGTCAGTG
Nipbl [NM_027707]	Forward<br>Reverse	5′-ATGCTCGGAACAAAGCAATTACCTC<br>5′-AGTGTCTACCCAAGGCACCAGTTTG
Vcp [NM_009503]	Forward<br>Reverse	5′-ACCCATCGATGACACAGTGGAAGGCA<br>5′-TCCTGCAACCACCGATGTCATCATAG
Brd1 [NM_001033274]	Forward<br>Reverse	5′-ACGAGTGCCTGGCCACCACAATGG<br>5′-GTCAATGTCACTGAGGTCACTGGC
Brd2 [NM_001204973]	Forward<br>Reverse	5′-GAAAGAAACCCCGGAAGCCCTACA<br>5′-TTAGCCCGAGTCTGAATCGCTGGT
Brd3 [NM_001113574]	Forward<br>Reverse	5′-TGAAGCCAACCACGCTGCGGGAAC<br>5′-TCACTCTGAGTCACTGCTGTCGGAG
Brd4 [NM_020508]	Forward<br>Reverse	5′-ATGAAGATGCGCTGGAACAGGC<br>5′-CAGAAGTCACCTGGGTGCTCTC
Brd7 [NM_001377016]	Forward<br>Reverse	5′-GATTGCTCAGGGAGCTCCAGGA<br>5′-GGCTAGTTCAGCTCGCGTCAGG
Brd8 [NM_030147]	Forward<br>Reverse	5′-CAGCGTGACATCATGCTGATGT<br>5′-TCTGCTTCAATGGCACAGCGGC
Brd9 [NM_001024508]	Forward<br>Reverse	5′-CCTGATGTCTCCCTGGATGTGTCC<br>5′-CTTGGCAGGAGCTGCAGGTTCTGG
Aicda [NM_009645]	Forward<br>Reverse	5′-TCTGGCTGCCACGTGGAATTGTTGTTCCTA<br>5′-TCAAAATCCCAACATACGAAATGCATCTCG
Shld2 [NM_001360074]	Forward<br>Reverse	5′-TTCTGTGGAGGACTGCAGCATTTG<br>5′-AGTACTGTCTCACCAATCCACTGAT
Hprt [NM_013556]	Forward<br>Reverse	5'-CTCGAAGTGTTGGATACAGG<br>5'-TGGCCTATAGGCTCATAGTG
**Sμ-Sα Junction**		
Sμ-Sα (1st PCR)	Sμ (1st PCR)-F<br>Sα (1st PCR)-R	5′-ATTCCACACAAAGACTCTGGACC<br>5′-AGCGCTCCAGATTTCTAAGCCCCACTCCTG
Sμ-Sα (2nd PCR)	Sμ (2nd PCR)-F<br>Sα (2nd PCR)-R	5′-GTAAGGAGGGACCCAGGCTAAG<br>5′-TTTGGGCAGTGGATAGAGCTATGTTCTCAG
**IgH/c-Myc Translocation**		
Sμ/c-Myc (1st PCR)	Sμ (1st PCR)-F<br>c-Myc (1st PCR)-R	5′-ACTATGCTATGGACTACTGGGGTCAAG<br>5′-GTGAAAACCGACTGTGGCCCTGGAA
Sμ/c-Myc (2nd PCR)	Sμ (2nd PCR)-F<br>c-Myc (2nd PCR)-R	5′-CCTCAGTCACCGTCTCCTCAGGTA<br>5′-GTGGAGGTGTATGGGGTGTAGAC
	c-Myc Probe	5′DIG-GGACTGCGCAGGGAGACCTACAGGGG
**LMPCR**	Linker-long	5′-GCGGTGACCCGGGAGATCTGAATTCAC
	Linker-short	5′-GTGAATTCAGATC
	Sμ-F	5′-GCAGAAAATTTAGATAAAATGGATACCTCAGTGG
	Linker-R	5′-GCGGTGACCCGGGAGATCTGAATTC
	Gapdh-F	5′-ATCCTGTAGGCCAGGTGATG
	Gapdh-R	5′-GCTCAAGGGCTTTTAAGGCT

**ChIP-qPCR (IgH)**	a-IgHμ-F	5'-AAAGAGACATTTGTGTGTCTTTGAGTACCG
	a-IgHμ-R	5'-ATTGGTTAACAGGCAACATTTTTCTTTTAC
	b-IgHμ-F	5'-TCTCTACTTCAGTTATACATGTGGGTTTGA
	b-IgHμ-R	5'-ATTAAAAACCACTGAGGTATCCATTTTATC
	c-IgHμ-F	5'-CAGCACCATTTCCTTCACCTGGAACTACCA
	c-IgHμ-R	5'-GGCTAGGTACTTGCCCCCTGTCCTCAGTGT
	d-IgHα-F	5'-TGAAAAGACTTTGGATGAAATGTGAACCAA
	d-IgHα-R	5'-GATACTAGGTTGCATGGCTCCATTCACACA
	e-IgHα-F	5'-TGGAGTGGTGGGTCTGAAGCCAGAACAAGG
	e-IgHα-R	5'-ACACTGGCGGGGCAGGGGGTTGTGTGTGGG
	f-IgHα-F	5'-AGTGCCCAGAAGGAGAATCCGTGAAATGTT
	f-IgHα-R	5'-GACCCCCTAACGTTCTTTACCAGAGCAATT
	Iμ-F	5'-AAGGGCTTCTAAGCCAGTCC
	Iμ-R	5'-CACAACCATACATTCCCAGGT
	Iα-F	5'-GTGATTCAGGGAGCAAGAGC
	Iα-R	5'-TCTAGCCTGGGAGTCTCCTG
**DNA end resection qPCR**	p1-F	5'-GAGCAATGTTGAGTTGGAGTCA
	p1-R	5'-GGCTGGACAGAGTGTTTCAAAA
	p2-F	5'-ACACAAAGACTCTGGACCTCTC
	p2-R	5'-GACTCAGTCCCTCTTAAGCAGA
	p3-F	5'-CCAGCCACAGTAATGACCCA
	p3-R	5'-AAGCACTCAGAGAAGCCCAC
	p4-F	5'-TGCAGGTCATGTTCAAAGTCT
	p4-R	5'-ACTGCCCATCTCGAGACAAT
	p5-F	5'-TGAGATCCTGCTACCACGGA
	p5-R	5'-TCATTGTACCCAGCTCCCAG
**ChIP-qPCR (I-*Sce*I site)**		
DSB1 (Left)	Forward<br>Reverse	5′-ACTATAGGGAGACCCAAGCTTGGTA<br>5′-CGAATCCGGAGACGTACGACCGGT
DSB2 (Right-1)	Forward<br>Reverse	5′-CGAGTGCCGCATCACCGGCACCCTG<br>5′-TTCATCTTGTTGGTCATGCGGCCCT
DSB2 (Right-2)	Forward<br>Reverse	5′-AATTCGTTAGGCCATTAAGGCCTGT<br>5′-TGCCGGTGATGCGGCACTCGATCTT
**3C PCR**		
Eμ-3'RR	Forward<br>Reverse	5'-GCTGACATGGATTATGTGAGG<br>5'-CAAGGTGTTAAGGAAAACTTGCTC
Sμ-Sα	Forward<br>Reverse	5'-GCTGACATGGATTATGTGAGG<br>5'-GCCTAGCCCAGACCATGCCA
Gapdh	Forward<br>Reverse	5'-CAGTAGACTCCACGACATAC<br>5'-AGTAGTGCGTTCTGTAGATTCC
***I-Sce*I Junction PCR**	NHEJ-F<br>NHEJ-R	5'-GTACGGTGGGAGGTCTATATAAG<br>5'-TTCATCTTGTTGTTCATGCGG
**Knockdown**	**Genes**	**Thermo Fisher (siRNAs)**
	Brd2 (Mouse)	5'-CCCAUCAGUCAUCUCUUCUCCUCUU(Custom)
	Brd2 (Human)	HSS184316, HSS184317
	Brd3 (Mouse)	MSS228281, MSS228282, MSS228283
	Brd4 (Mouse)	MSS226481

	Vcp/p97 (Mouse)	MSS219083, **MSS219084**, MSS219085
	Nipbl (Mouse)	MSS230655, **MSS230656**, MSS230657
	Shld2 (Mouse)	5'-CCUGUGUAUGUCUGUGUACCAUGUA (Custom)
	Control siRNA	# 12935-300
**CRISPR/Cas9 Targeting**	**Guide RNA**	**Sequence**
	Sμ-Specific	5′-GTTGAGAGCCCTAGTAAGCG agg(pam)
	Sα-Specific	5′-GCTTGGAAGTTACACTGGCG ggg(pam)

Table S3

Sequences of Sμ-Sα recombination junctions from CH12F3-2A cells transfected with siControl. The bar (|) indicates a direct or blunt Sμ-Sα junction, the boldface shows microhomology or nucleotide overlap at the junction, and the underline indicates nucleotide insertion.

Control (n = 41)

Sμ Sα

AATAGAGACCTGCAGTTGAGGCCAGCAGGT|TTAGGCGAAACTTGGCTTGGCTGGTTACAA

GGTTGTTAAAGAATGGTATCAAAGGACAGT|CGTGCTGGCTGATCTTCAGTCTCAGGTTGG

AAGGCAATCCTGGGATTCTGGAAGAAAAGA|TCCCCAACCACTCCTACCCCGTGCTGGCTG

AGGCTGGGCTGAGCTGGAATGAGCTGGGTT|TGGAGTGGTGGGTCTGAAGCCAGAACAAGG

TTAGTTTTTATAGAAAACACTACTACATTC|CTGGTGGAAATTCACATGTACCTGTAAATG

AATTAAGGGAACAAGGTTGAGAGCCCTAGT|TGTACCTGTAAATGGTGCCTTCATGGCAGG

GTGTGAGCTAGACTGAGCTGAACTAGGGTG|CAGCTGGCTGAACCAAACTTGACAGTGAGC

AGGCTGGGCTGAGCTGGAATGAGCTGGGTT|TGGAGTGGTGGGTCTGAAGCCAGAACAAGG

CTTCCTGGTTGTTAAAGAATGGTATCAAAG|ATTAGGCGAAACTTGGCTTGGCTGGTTACA

AATGGATTGAGCTGAGCTAGACTAGGGTGG|GCTAGCCTGGGGTGAATTAGCATGACTGGA

GAAGGCAATCCTGGGATTCTGGAAGAAAAG|TCCCCAACCACTCCTACCCCGTGCTGGCTG

TCCAAGGTGAGTGTGAGAGGACAGGGGCTG|GGGATGGGATGGGATGAACTGACTGGGCTG

ATCCAAGGTGAGTGTGAGAGGACAGGGGCT|CAAACTTGACAGTGAGCTAGCCTGGGGTGA

AAAAAGCATGGCTGAGCTGAGATGGGTGGG|ACTGAAAAGACTTTGGATGAAATGTGAACC

AGAATTGAGAAAGAATAGAGACCTGCAGTT|TGAGAACATAGCTCTATCCACTGCCCAAAC

GACTGTTCTGAGCTGAGATGAGCTGGGGTG**A**CTAGTATAAACTTGGCTAGGCTACAATGGA

AGAGCCCTAGTAAGCGAGGCTCTAAAAAGC**A**CATAAATTCAGCTGGCTGAACCAAACTTGA

AGTGTGAGAGGACAGGGGCTGGGGTATGGA**T**TGGTTTGCTTTGTGTGAATGGAGCCATGC-

CGCAGAAGGAAGGCCACAGCTGTACAGAAT**T**AGGCTGGAATTAGGCGAAACTTGGCTTGGC

GGCCAGACTCATAAAGCTTGCTGAGCAAAA**T**GAACCAAACTTGACAGTGAGCTAGCCTGGG

GCTGTACAGAATTGAGAAAGAATAGAGACC**T**CTGGTCCTCCTAACCCTGGGACTGAGAACA

GGGAACAAGGTTGAGAGCCCTAGTAAGCGA**G**TTTGCTTTGTGTGAATGGAGCCATGCAACC

GGGGCTGGGGTATGGATACGCAGAAGGAAG**G**TGTGAATGGAGCCATGCAACCTAGTATCCT

CTGAGCTGAGCTGGGCTAAGCTGGGATGGA**C**CTGGGGTGAATTAGCATGACTGGACTTAT-

TGTTGGGGTGAGCTGATCTGAAATGAGCTA**C**ACATGTACCTGTAAATGGTGCCTTCATGGC

GTTGAATAGAGCTAAATTCTACTGCCTACA**CT**CAGGTTGGCCACCCCTGCCCAGACCCACCA

ACTAGGCTGGCTTAACCGAGATGAGCCAAA**CT**CAGATTGAGTCCTCTCGTCTTTGGGTCCAC

GGTGAGTGTGAGAGGACAGGGGCTGGGGTA**TG**AACTGACTGGGCTGGACTCAGTTGACCTTG

GAATTGAGAAAGAATAGAGACCTGCAGTTG**AG**TTGGGCTGGCCAGGATAGTCAGAACTAGGC

GAACAAGGTTGAGAGCCCTAGTAAGCGAGG**CT**GGACTTATTCACAGTTCTAGCCTGAGCTTT

ACAGTAATGACCCAGACAGAGAAGGCCAGA**CT**AGCCTGAGCTTTGCTGGATTGTTAAACTCA

AGAATTGAGAAAGAATAGAGACCTGCAGTT**GA**CTTATTCACAGTTCTAGCCTGAGCTTTGC-

GACCCAGACAGAGAAGGCCAGACTCATAAA**CCT**TGGTTTGCTTTGTGTGAATGGAGCCATGCA

TTTTAGTTTTTATAGAAAACACTACTACAT**TCT**GGTGGAAATTCACATGTACCTGTAAATGGT

GTGGAATGGGCTGAACTAGGGTGAACTGGG**CTGA**GCTTTGCTGGATTGTTAAACTCACTGCTGA

TGTACAGAATTGAGAAAGAATAGAGACCTG**CAGTT**CTGGTCCTCCTAACCCTGGGACTGAGAACA

TTAGATCCAAGGTGAGTGTGAGAGGACAGG**GGCTGG**GGTTAGGCGAAACTTGGCTTGGCTGGTTAC

GAGCAAAATTAAGGGAACAAGGTTGAGAGC**CCTAGTA**TATCCTATTCTCACACCCCTCCTCCTTCCC

GGGCTGGGGTATGGATACGCAGAAGGAAGGTCAAACTTGACAGTGAGCTAGCCTGGGGTGA

TGAGAGCCCTAGTAAGCGAGGCTCTAAAAAACACAACTTGACAGTGAGCTAGCCTGGGGTGAAT

GGGGTATGGATACGCAGAAGGAAGGCCACAIn29ATTAGGCGAAACTTGGCTTGGCTGGTTACA

Table S3 (Cont.)

Sequences of Sμ-Sα recombination junctions from CH12F3-2A cells transfected with siBrd2. The bar (|) indicates a direct or blunt Sμ-Sα junction, the boldface shows microhomology or nucleotide overlap at the junction, and the underline indicates nucleotide insertion.

Brd2 knockdown (n = 34)

Sμ Sα

CAATGTGGTTTAATGAATTTGAAGTTGCCA|AGCCTGGGGTGAATTAGCATGACTGGACTT

AACTCAATGTGGTTTAATGAATTTGAAGTT|CTGTCCCGCCAGTGTAACTTCCAAGCCAGC

GAGGTAAACAAAGCTGGGCTTGAGCCAAAA|GGCCACCTGCCCAGACCCACCAGTTCTGGT

TGTTCTGAACTGAGATGAGCTGGGGTGAGC|GCCCAGACCCACCAGTTCTGGTCCTCCTAA

TGAGCTGAACTGGGCTGAGTTAGACTGAGC**T**ACAATGAGCTAACATAAATTCAGCTGGCTG

GCTGGGATGGACTAGGATAAACTAAGCTGG**G**GTTGGCCACCCCTGCCCAGACCCACCAGTT

CCACAGTAATGACCCAGACAGAGAAAGCCA**GA**TCTTCAGTCTCAGGTTGGCCACCCCTGCCC

GGGGCTGGGGTATGGATACGCAGAAGGAAG**GC**TTTGCTGGATTGTTAAACTCACTGCTGAGG

ATTAAGGGAACAAGGTTGAGAGCCCTAGTA**AGC**TGGGATGGGATGGGATGGGATGGGATGGGA

TACGCGTGTTGGGGTGAGCTGATCTGAAAT**GAG**ATTCACATGTACCTGTAAATGGTGCCTTCA

ACAGTTGTACAGAATTGAGAAAGAATAGAG**ACC**AGTTCTGGTCCTCCTAACCCTGGGACTGAG

AGCTGGGATGGACTAGGATAAACTAAGCTG**GGTTG**GCCACCCCTGCCCAGACCCACCAGTTCTGG

TGTACAGAATTGAGAAAGAATAAGACCTA**CAGTT**CTGGTCCTCCTAACCCTGGGACTGAGAACA

TTAGATCCAAGGTGAGTGTGAGAGGACAGG**GGCTG**AACCAAAGAATACAGTGAGCTAGCCTGGGG

CACAGCTGTACAGAATTGAGAAAGAATAG**AGACC**TACCAGTTCTGGTCCTCCTAACCCTGGGAC

TGTACAGAATTGAGAAAGAATAAGACCTA**CAGTT**CTGGTCCTCCTAACCCTGGGACTGAGAACA

TGTACAGAATTGAGAAAGAATAAGACCTG**CAGTT**CTGGTCCTCCTAACCCTGGGACTGAGAACA

CTGGGCCGCTAAGCTAAACTAGGCTGGCTT**AACCTAG**TATCCTATTCTCACACCCCCTCCTCCTTCC

TACTTCCTGGTTGTTAAAGAATGGTATCAA**AGGACAG**AGACACCGCTCAGATTGAGTCCTCTCGTCT

GGCTGGGGTATGGATACGCAGAAGGAAGGC**AGACCTA**CAGTTCTGGTCCTCCTAACCCTGGGACTGA

TGTTGGGGTGAGCTGATCTGAAAT**GAGATAC**ACATGTACCTGTAAATGGTGCCTTCATGGC

GTTGCCAGTAAATGTACTTCCTGGTTGTTATCTGGGACTGAGAACATAGCTCTATCCACTG

TTCTGAGCTGGGGTGAACTGGGGTGAGCTGATCACATGTACCTGTAAATGGTGCCTGCATG

GCGTGTTGGGGTGAGCTGATCTGAAATGAGAATCACATGTACCTGTAAATGGTGCCTGCATG

GGTTGAGAGCCCTAGTAAGCGAGGCTCTAAGTTGCTGAGGTAACTGAAAAGACTTTGGATGA

CCTGGGATTCTGGAAGAAAAGATGTTTTTACTAGGTCTTCCCCAACCACTCCTACCCCGTGC

TACAGAATTGAGAAAGAATAGAGACCTGCACTGAGCTGAGTTGAGCTGAACCAAACTTGACAGTGAGCTAGCCT

TACAGAATTGAGAAAGAATAGAGACCTGCAIn29GGTGAGTCTGAAGCCAGAACAAGGCCAGTG

GGCTTCTCTGAGCGCTTCTAAAATGCGCTAIn34GAAATTCACATGTACCTGTAAATGGTGCCT

AAGGGAACAAGGTTGAGAGCCCTAGTAAGCIn37GGATGGGATGGGATGGGATGAACTGACTGG

GGCTTCTCTGAGCGCTTCTAAAATGCGCTAIn37ATTCACATGTACCTGTAAATGGTGCCTTCA

AAGGGAACAAGGTTGAGAGCCCTAGTAAGCIn49GGTCCTTTTTCCTGAAATTGTCTCGAGATG

TAGATCCGAGGTGAGTGTGAGAGGACAGGGIn70AGGTAACTGAAAAGACTTTTGATGAAATGT

CCAGTAAATGTACTTCCTGGTTGTTAAAGAIn124ATGTACCTGTAAATGGTGCCTTCATGGCAG

Table S3 (Cont.)

Sequences of Sμ-Sα recombination junctions from CH12F3-2A cells transfected with siNipbl. The bar (|) indicates a direct or blunt Sμ-Sα junction, the boldface shows microhomology or nucleotide overlap at the junction, and the underline indicates nucleotide insertion.

Nipbl knockdown (n = 38)

Sμ Sα

ACAGTAATGACCCAGACAGAGAAAGCCAGA | GGACTGAGAACATAGCTCTATCCACTGCCC

TCGGCTGGACTAACTCTCCAGCCACAGTAA | AAATCACATGTACCTGTAAATGGTGCCTTC

TAAAATGCGCTAAACTGAGGTGATTACTCT | TGAGCTGAACTAGGATGGGATGGGATGGGA

TAAGCGAGGCTCTAAAAAGCATGGCTGAGC | ACCAACTCTTGTTTTATGGTCCAGATTGTG

GATGGGTGGGCTTCTCTGAGTGCTTCTAAA**A**GCCATGCAACCTAGTATCCTATTCTCACAC

AAATGAAGTAGACTGTAATGAACTGGAATG**A**CCCCGTGCTGGCTGATCTTCAGTCTCAGGT

CTGCAGTTGAGGCCAGCAGGTCGGCTGGAC**T**GAGCTAACATAAATTCAGCTGGCTGAACCA

GACAGAGAAGGCCAGACTCATAAAGCTTGC**T**TCTAGCCTGAGCTTTGCTGGATTGTTAAAC

TCATAAAGCTTGCTGAGTAAAATTAAGGGA**AC**CCCGTGCTGGCTGATCTTCAGTCTCAGGTT

GGCTTAACCGAGATGAGCCAAACTGGAATG**AA**AATGGCCTTCTCCACCCCACACACAACCCC

CTTCTAAAATGCGCTAAACTGAGGTGATTA**CT**AGTTGGGCTGGCCAGGATAGTCAGAACTAG

TGGAATGAGCTGGGCCGCTAAGCTAAACTA**GGC**AGGACAGAGACACCGCTCAGATTGAGTCCT

GAGCGCTTCTAAAATGCGCTAAACTGAGGT**GAT**GAACTGACTGGGCTGGACTCAGTTGACCTT

AAGAAAAGATGTTTTTAGTTTTTATAGAAA**ACAC**CCCCTCCTCCTTCCCTGATTACCTGGTGGA

GCTCAGCTATGCTACGCGTGTTGGGGTGAG**CTGA**AAAGACTTTGGATGAAATGTGAACCAACTC

CGCTAAGCTAAACTAGGCTGGCTTAACCGA**GATGA**ACTGACTGGGCTGGACTCAGTTGACCTTGC

AATTTGAAGTTGCCAGTAAATGTATTTCCT**GGTTG**CCAGAATAGTCAGAACTAGGCTGGAATTAG

CTGAGGTGATTACTCTGAGGTAAGCAAAGC**TGGCT**AGCCTGGGGTGAATTAGCATGACTGGACTT

GCGCTTCTAAAATGCGCTAAACTGAGGTGA**TTACTC**ACAGTTCTAGCCTGAGCTTTGCTGGATTGT

TGAGCTGAGCTAGGGTGAGCTGAGCTGGG**TGGCTGA**ACCAAACTTGACAGTGAGCTAGCCTGGGGT

TGTACAGAATTGAGAAAGAATAGAGACCTG**CAGTTGAG**TCCTCTCGTCTTTGGGTCCACTGCTTGCTA

CTGGGGTGAGCTCAGCTATGCTACGCGTGT**TGGGGTGA**ATTAGCATGACTGGACTTATTCACAGTTCT

AGCTGAGATGGGGTGAGATGGGGTGAGCTGA**GCTGGGCTGA**TTAGTCTAGGCTGGACCAAATTAGGCTGGA

CCACAGCTGTACAGAATTGAGAAAGAATAG**AGACCTGCAGTT**CTGGTCCTCCTAACCCTGGGACTGAGAACA

AGGGAACAAGGTTGAGAGCCCTAGTAAGCGTCTGGGGTGAATTAGCATGACTGGACTTATT

TAAGGGAACAAGGTTGAGAGCCCTAGTAAGGTCAGCTGGCTGAACCAAACTTGACAGTGAG

AGCTGGGTGAGCTGAGCTGAGCTGGGGTGAACCTCTGGTTTGCTTTGTGTGAATGGAGCCA

CTTAGATCCAAGGTGAGTGTGAGAGGACAGAAACTGGGATGGGATGGGATGGGATGGGATGGGA

TCGGCTGGACTAACTCTCCAGCCACAGTAAATCACATGTACCTGTGAATGGAGCCATGCAACCTAGTATCCT

TACAGAATTGAGAAAGAATAGAGACCTGCAIn50CTGAGCTGAGCTGGGCTAAGCTGGGATGGA

TTCTTGATCTACAACTCAATGTGGTTTAATIn20AATGGCCTTCTCCACCCCACACACAACCCC

GAGCTGGGCCGCTAAGCTAAACTAGGCTGGIn90TGCTCGTCTGAGCTGGTCTAGATGGTCTAG

AGAGCCCTAGTAAGCGAGGCTCTAAAAAGCIn65GGATGGGATGGGATGGGATGGGATGGGATG

AATGCGCTAAACTGAGGTGATTACTCTGAGIn68GGATGGGATGGGATGGGATGGGATGGGATG

CATAAAGCTTGCTGAGCAAAATTAAGGGAAIn20TTAGCATGACTGGACTTATTCACAGTTCTA

TGGTATCAAAGGACAGTGCTTAGATCCAAGIn378TGTGTGAATGGAGCCATGCAACCTAGTATC

GTATCAAAGGACAGTGCTTAGATCCAAGGTIn106GTGTGAATGGAGCCATGCAACCTAGTATCC

GGGTGAGCTGAGCTGAGCTGGGGTGAGCTGIn119TGCTCGTCTGAGCTGGTCTAGATGGTCTAG

Supplementary Table S4. MiQE Checklist for RT-qPCR

ITEM TO CHECK	IMPORTANCE	CHECKLIST
**EXPERIMENTAL DESIGN**		
Definition of experimental and control groups	E	RT-qPCRs were carried out for GLT and the gene knocked down with reference to housekeeping gene control.
Number within each group	E	3 in most of the cases.
Assay carried out by core lab or investigator's lab?	D	
Acknowledgement of authors' contributions	D	
**SAMPLE**		
Description	E	RNAs were extracted from CH12 and primary B cells.
Volume/mass of sample processed	D	CH12 : ~5 -10 x10[5] cells; Primary B cells: ~1- 2 x10[5] cells
Microdissection or macrodissection	E	n/a
Processing procedure	E	Stored at -80C
If frozen - how and how quickly?	E	Immidiate after preparation or quantitation.
If fixed - with what, how quickly?	E	n/a
Sample storage conditions and duration (especially for FFPE sa	E	n/a
**NUCLEIC ACID EXTRACTION**		
Procedure and/or instrumentation	E	Total RNA extraction from B cells
Name of kit and details of any modifications	E	TRIzol Reagent (Thermo Fisher Scientific) and the manufacture's protocol
Source of additional reagents used	D	
Details of DNase or RNAse treatment	E	RNeasy MinElute Cleanup Kit (QIAGEN) was used to clean up RNA after DNase treatment.
Contamination assessment (DNA or RNA)	E	RT(-) control was conducted during RT-qPCR.
Nucleic acid quantification	E	Total RNAswere quantified.
Instrument and method	E	NanoDrop Spectrophotometer (Thermo Fisher Scientific)
Purity (A260/A280)	D	
Yield	D	
RNA integrity method/instrument	E	2100 Bioanalyzer System (Agilent)
RIN/RQI or Cq of 3' and 5' transcripts	E	RIN 8-10
Electrophoresis traces	D	
Inhibition testing (Cq dilutions, spike or other)	E	Not done.
**REVERSE TRANSCRIPTION**		
Complete reaction conditions	E	cDNA sythesis using SuperScript® IV Reverse Transcriptase (Thermo Fisher Scientific)
Amount of RNA and reaction volume	E	1 μg /20 μl
Priming oligonucleotide (if using GSP) and concentration	E	Oligo dT
Reverse transcriptase and concentration	E	SuperScript® IV RT
Temperature and time	E	50°C for 1 h
Manufacturer of reagents and catalogue numbers	D	
Cqs with and without RT	D	
Storage conditions of cDNA	D	
**qPCR TARGET INFORMATION**		
Gene symbol	E	Indicated in Supplementary Table S2
Sequence accession number	E	See Supplementary Table S2
Location of amplicon	D	
Amplicon length	E	200-300 bp
In silico specificity screen (BLAST, etc)	E	BLASTn
Pseudogenes, retropseudogenes or other homologs?	D	
Sequence alignment	D	
Secondary structure analysis of amplicon	D	
Location of each primer by exon or intron (if applicable)	E	Primers are loacted on the exon.
What splice variants are targeted?	E	No splice variant was trageted.
**qPCR OLIGONUCLEOTIDES**		
Primer sequences	E	Reported in Supplementary Table S2
RTPrimerDB Identification Number	D	
Probe sequences	D	
Location and identity of any modifications	E	n/a
Manufacturer of oligonucleotides	D	
Purification method	D	
**qPCR PROTOCOL**		
Complete reaction conditions	E	Standard reaction conditions according to manual of PowerUp™ SYBR™ Green Master Mix (Applied Biosystems/ Thermo Fisher Scientific)
Reaction volume and amount of cDNA/DNA	E	10 µl reaction with 0.1 µl cDNA/ reaction
Primer, (probe), Mg++ and dNTP concentrations	E	0.5 μM, 2X master mix of PowerUp™ SYBR™ Green
Polymerase identity and concentration	E	Dual-Lock Taq DNA Polymerase (Thermo Fisher Scientific)
Buffer/kit identity and manufacturer	E	Applied Biosystems/Thermo Fisher Scientific Master Mix Cat # A25742
Exact chemical constitution of the buffer	D	
Additives (SYBR Green I, DMSO, etc.)	E	SYBR™ Green Master Mix above.
Manufacturer of plates/tubes and catalog number	D	
Complete thermocycling parameters	E	Hot start, followed by 40 cycles of denaturation (95°C for 15 sec) and anealing-extension (60°C for 1 min).
Reaction setup (manual/robotic)	D	Manual
Manufacturer of qPCR instrument	E	Applied Biosystems Quanta Studio 5 Real Time PCR System
**qPCR VALIDATION**		
Evidence of optimisation (from gradients)	D	
Specificity (gel, sequence, melt, or digest)	E	Determined by Melt Curve analysis.
For SYBR Green I, Cq of the NTC	E	At or below the detection limit.
Standard curves with slope and y-intercept	E	Standard curves were examined by real-time qPCR using serially diluted cDNA as a template.
PCR efficiency calculated from slope	E	PCR efficiency was greater than 95%.
Confidence interval for PCR efficiency or standard error	D	
r2 of standard curve	E	r2 value>0.95
Linear dynamic range	E	Sample detection was within the values in the standard curve.
Cq variation at lower limit	E	All assays were performed within the linear range of the standard curve.
Confidence intervals throughout range	D	
Evidence for limit of detection	E	All assays were performed within the linear range of the standard curve.
If multiplex, efficiency and LOD of each assay.	E	n/a
**DATA ANALYSIS**		
qPCR analysis program (source, version)	E	Quanta Studio Design2 (Applied Biosystems); Excel 2016
Cq method determination	E	Quanta Studio Design2 (Applied Biosystems)
Outlier identification and disposition	E	n/a
Results of NTCs	E	At or below the detection limit in at least 40 cycles.
Justification of number and choice of reference genes	E	*Hprt* gene used as a reference.
Description of normalisation method	E	Cq values are nornalized with those of housekeeping control gene
Number and concordance of biological replicates	D	
Number and stage (RT or qPCR) of technical replicates	E	2 or 3
Repeatability (intra-assay variation)	E	Standard deviation check
Reproducibility (inter-assay variation, %CV)	D	
Power analysis	D	
Statistical methods for result significance	E	t-test p value
Software (source, version)	E	Excel 2016
Cq or raw data submission using RDML	D	

MIQE checklist for authors, reviewers and editors. All essential information (E) must be submitted with the manuscript. Desirable information (D) should be submitted if available. If usi primers obtained from RTPrimerDB, information on qPCR target, oligonucleotides, protocols and validation is available from that source.

*: Assessing the absence of DNA using a no RT assay is essential when first extracting RNA. Once the sample has been validated as RDNA-free, inclusion of a no-RT control is desira but no longer essential.

**: Disclosure of the probe sequence is highly desirable and strongly encouraged. However, since not all commercial pre-designed assay vendors provide this information, it cannot b essential requirement. Use of such assays is advised against.

Supplementary Table S5. MiQE Checklist for qPCR related to ChIP, DRIP, and DNA end-resection assay

ITEM TO CHECK	IMPORTANCE	CHECKLIST
**EXPERIMENTAL DESIGN**		
Definition of experimental and control groups	E	Described in the respective method section in the manuscript
Number within each group	E	3 or more
Assay carried out by core lab or investigator's lab?	D	
Acknowledgement of authors' contributions	D	
**SAMPLE**		
Description	E	CH12F3-2A cells
Volume/mass of sample processed	D	
Microdissection or macrodissection	E	n/a
Processing procedure	E	Stored at -80C (chromatin); DNA at -20C or 4C (immidiate use)
If frozen - how and how quickly?	E	n/a
If fixed - with what, how quickly?	E	n/a
Sample storage conditions and duration (especially for FFPE sa	E	n/a
**NUCLEIC ACID EXTRACTION**		
Procedure and/or instrumentation	E	DNA was prepared following IP or without IP as may require.
Name of kit and details of any modifications	E	ChIP assay: Active Motif ChIP Kit & following the instruction. DNA extraction by Pheno l: Chlorofor
Source of additional reagents used	D	
Details of DNase or RNAse treatment	E	ChIP: RNase A. DRIP : RNase A & RNaseH (-/+)
Contamination assessment (DNA or RNA)	E	n/a
Nucleic acid quantification	E	Purified DNA was quantified as necessary
Instrument and method	E	NanoDrop Spectrophotometer (Thermo Fisher Scientific)
Purity (A260/A280)	D	
Yield	D	
RNA integrity method/instrument	E	n/a
RIN/RQI or Cq of 3' and 5' transcripts	E	n/a
Electrophoresis traces	D	
Inhibition testing (Cq dilutions, spike or other)	E	Not done.
**REVERSE TRANSCRIPTION**		
Complete reaction conditions	E	n/a
Amount of RNA and reaction volume	E	n/a
Priming oligonucleotide (if using GSP) and concentration	E	n/a
Reverse transcriptase and concentration	E	n/a
Temperature and time	E	n/a
Manufacturer of reagents and catalogue numbers	D	
Cqs with and without RT	D	
Storage conditions of cDNA	D	
**qPCR TARGET INFORMATION**		
Gene symbol	E	n/a
Sequence accession number	E	Mouse IgH locus (Chr 12 - NC_000078.7) Sμ: AH005309 and Sα: D11468.1
Location of amplicon	D	
Amplicon length	E	200-300 bp
In silico specificity screen (BLAST, etc)	E	BLASTn
Pseudogenes, retropseudogenes or other homologs?	D	
Sequence alignment	D	
Secondary structure analysis of amplicon	D	
Location of each primer by exon or intron (if applicable)	E	Illustrated in the Figure
What splice variants are targeted?	E	n/a
**qPCR OLIGONUCLEOTIDES**		
Primer sequences	E	Reported in Supplementary Table S2
RTPrimerDB Identification Number	D	
Probe sequences	D	
Location and identity of any modifications	E	None
Manufacturer of oligonucleotides	D	
Purification method	D	
**qPCR PROTOCOL**		
Complete reaction conditions	E	Standard reaction conditions according to manual of PowerUp™ SYBR™ Green Master Mix (Applied Biosystems/ Thermo Fisher Scientific)
Reaction volume and amount of cDNA/DNA	E	10 µl reaction with 0.5 ul DNA per reaction.
Primer, (probe), Mg++ and dNTP concentrations	E	0.5 µM, 2X master mix of PowerUp™ SYBR™ Green
Polymerase identity and concentration	E	Dual-Lock Taq DNA Polymerase (Thermo Fisher Scientific)
Buffer/kit identity and manufacturer	E	Applied Biosystems/Thermo Fisher Scientific Master Mix Cat # A25742
Exact chemical constitution of the buffer	D	
Additives (SYBR Green I, DMSO, etc.)	E	SYBR™ Green Master Mix above.
Manufacturer of plates/tubes and catalog number	D	
Complete thermocycling parameters	E	Hot start, followed by 40 cycles of denaturation (95°C for 15 sec) and anealing-extension (60°C for 1 min).
Reaction setup (manual/robotic)	D	Manual
Manufacturer of qPCR instrument	E	Quanta Studio 5 or 7900HT Real Time PCR System (Applied Biosystems)
**qPCR VALIDATION**		
Evidence of optimisation (from gradients)	D	
Specificity (gel, sequence, melt, or digest)	E	Melt Curve analysis.
For SYBR Green I, Cq of the NTC	E	At or below the detection limit.
Standard curves with slope and y-intercept	E	Standard curves were examined by real-time qPCR using serially diluted input DNA.
PCR efficiency calculated from slope	E	PCR efficiency was greater than 95%.
Confidence interval for PCR efficiency or standard error	D	
r2 of standard curve	E	r2 value>0.95
Linear dynamic range	E	Sample detection was within the values in the standard curve.
Cq variation at lower limit	E	All assays were performed within the linear range of the standard curve.
Confidence intervals throughout range	D	
Evidence for limit of detection	E	All assays were performed within the linear range of the standard curve.
If multiplex, efficiency and LOD of each assay.	E	n/a
**DATA ANALYSIS**		
qPCR analysis program (source, version)	E	Quanta Studio Design2 (Applied Biosystems); Excel 2016
Cq method determination	E	Quanta Studio Design2 (Applied Biosystems)
Outlier identification and disposition	E	n/a
Results of NTCs	E	At or below the detection limit in at least 40 cycles.
Justification of number and choice of reference genes	E	*Hprt* gene used as a reference.
Description of normalisation method	E	Standard curve quantification
Number and concordance of biological replicates	D	
Number and stage (RT or qPCR) of technical replicates	E	2 or 3
Repeatability (intra-assay variation)	E	Standard deviation check
Reproducibility (inter-assay variation, %CV)	D	
Power analysis	D	
Statistical methods for result significance	E	t-test p value
Software (source, version)	E	Excel 2016
Cq or raw data submission using RDML	D	

MIQE checklist for authors, reviewers and editors. All essential information (E) must be submitted with the manuscript. Desirable information (D) should be submitted if available. If usi primers obtained from RTPrimerDB, information on qPCR target, oligonucleotides, protocols and validation is available from that source.

*: Assessing the absence of DNA using a no RT assay is essential when first extracting RNA. Once the sample has been validated as RDNA-free, inclusion of a no-RT control is desira but no longer essential.

**: Disclosure of the probe sequence is highly desirable and strongly encouraged. However, since not all commercial pre-designed assay vendors provide this information, it cannot b essential requirement. Use of such assays is advised against.
